# *n*-Butanol production by *Rhodopseudomonas palustris* TIE-1

**DOI:** 10.1038/s42003-021-02781-z

**Published:** 2021-11-03

**Authors:** Wei Bai, Tahina Onina Ranaivoarisoa, Rajesh Singh, Karthikeyan Rengasamy, Arpita Bose

**Affiliations:** 1 Department of Energy, Environmental and Chemical Engineering, Washington University in St. Louis, St. Louis, MO USA; 2 Department of Biology, Washington University in St. Louis, St. Louis, MO USA

**Keywords:** Metabolic engineering, Applied microbiology

## Abstract

Anthropogenic carbon dioxide (CO_2_) release in the atmosphere from fossil fuel combustion has inspired scientists to study CO_2_ to biofuel conversion. Oxygenic phototrophs such as cyanobacteria have been used to produce biofuels using CO_2_. However, oxygen generation during oxygenic photosynthesis adversely affects biofuel production efficiency. To produce *n*-butanol (biofuel) from CO_2_, here we introduce an *n*-butanol biosynthesis pathway into an anoxygenic (non-oxygen evolving) photoautotroph, *Rhodopseudomonas palustris* TIE-1 (TIE-1). Using different carbon, nitrogen, and electron sources, we achieve *n*-butanol production in wild-type TIE-1 and mutants lacking electron-consuming (nitrogen-fixing) or acetyl-CoA-consuming (polyhydroxybutyrate and glycogen synthesis) pathways. The mutant lacking the nitrogen-fixing pathway produce the highest *n*-butanol. Coupled with novel hybrid bioelectrochemical platforms, this mutant produces *n*-butanol using CO_2_, solar panel-generated electricity, and light with high electrical energy conversion efficiency. Overall, this approach showcases TIE-1 as an attractive microbial chassis for carbon-neutral *n-*butanol bioproduction using sustainable, renewable, and abundant resources.

## Abstract (web summary)

Bai et al show that the anoxygenic phototroph strain TIE-1 can produce *n*-butanol using organic acids or CO_2_ as a carbon source, light as an energy source, and H_2_, Fe(II), or electrons from renewably generated electricity as an electron source. This study opens the possibility of producing carbon-neutral biofuels using these bacteria.

## Introduction

**Fig. 1:**
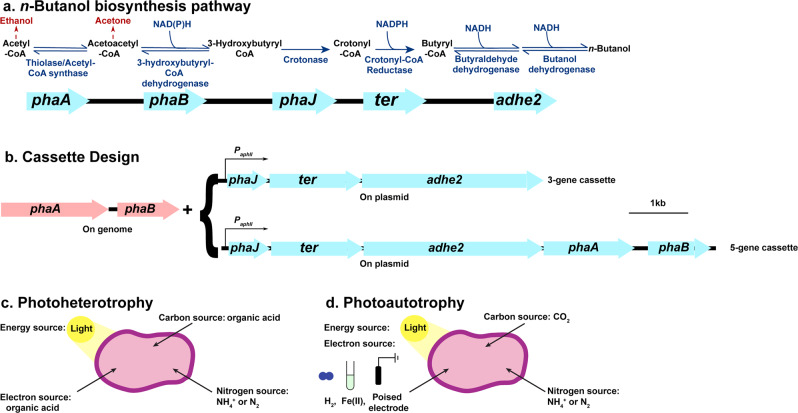
*n*-Butanol biosynthesis pathway, cassette design, and major metabolisms used for *n*-butanol production in *Rhodopseudomonas palustris* TIE-1 (TIE-1). **a** *n*-butanol biosynthesis pathway involves five genes. The enzymes encoded by each gene and the reactions catalyzed by these enzymes are shown in dark blue. Two major byproducts (acetone and ethanol) are shown in dark red. NADH, nicotinamide adenine dinucleotide; NADPH, nicotinamide adenine dinucleotide phosphate. **b** Cassette design. The 3-gene cassette relies on *phaA* and *phaB* on the genome of TIE-1 for the first two steps of *n*-butanol synthesis. Here, only 3-genes (*phaJ*, *ter*, and *adhE*2) were introduced on a plasmid under a constitutive promoter P_aphII_. The 5-gene cassette has all five genes (*phaA*, *phaB*, *phaJ*, *ter*, and *adhE*2) on the plasmid under a constitutive promoter P_aphII_. **c** Photoheterotrophy: TIE-1 uses organic acids as carbon and electron source, light as an energy source, and ammonium (NH_4_^+^) or dinitrogen gas (N_2_) as a nitrogen source. **d** Photoautotrophy: TIE-1 uses carbon dioxide as carbon source, hydrogen (H_2_), ferrous iron [Fe(II)], or poised electrode as an electron source, light as an energy source, and NH_4_^+^ or N_2_ as a nitrogen source.

The rapid consumption of fossil fuels has increased carbon dioxide (CO_2_) levels in the atmosphere raising concerns about global warming^1,2^. This has spurred research initiatives aiming to develop carbon-neutral biofuels that, when burned, will not result in net CO_2_ release^3^. Among the various biofuels, *n*-butanol has received greater attention due to its higher energy content, lower volatility, and reduced hydrophilicity compared to ethanol^4^. Currently, most *n*-butanol is synthesized via chemical processes^5,6^. However, these processes use propylene or ethanol as feedstocks, making these methods carbon-positive^5,6^. Another well-known strategy for *n*-butanol production is the acetone–butanol–ethanol (ABE) fermentation using *Clostridium* species^7^. The *n*-butanol biosynthesis pathway^7^ (Fig. 1a) from *Clostridium acetobutylicum* has been introduced into several organisms, such as *Escherichia coli*, *Saccharomy*ces *cerevisiae*, *Pseudomonas putida*, and *Bacillus subtilis* for *n*-butanol production^8–11^. However, most of these organisms are chemoheterotrophs. Thus, the *n*-butanol production using these microbes is also carbon-positive.

To date, only a handful of studies have produced *n*-butanol autotrophically using CO_2_ as a carbon source^12–16^. Using a microbial electrosynthesis approach, chemoautotroph *Clostridium* spp. produced 135 mg/L of *n*-butanol at an applied potential (*E*_appl_) of 0.8 V using CO_2_ in 35 days^12^. This prolonged period was required for acid accumulation for *n*-butanol production using *Clostridium* spp.^7^. Autotrophic *n*-butanol production was also demonstrated by an oxygenic photoautotroph *Synechococcus elongatus* PCC 7942 using water as an electron donor^14^ and sunlight as the energy source. Because the *n*-butanol was generated using solar energy, this product is called a solar fuel. With the *n*-butanol biosynthesis pathway, *S. elongatus* produced 2.2 mg/L *n*-butanol^14^ when incubated anaerobically under illumination. In contrast, aerobic incubation did not generate any *n*-butanol. Furthermore, a dark anaerobic incubation of dense cultures (where cells were not actively growing or evolving oxygen) produced 14 mg/L of *n*-butanol^14^. These results suggest that oxygen (O_2_) is detrimental to *n*-butanol production^14^. The ability of cyanobacteria to produce *n*-butanol was later improved by several modifications such as (1) using cofactor as a driving force^15^; (2) replacing the oxygen-sensitive enzyme involved in the *n*-butanol producing pathway^16^, and (3) using intensive genetic engineering to optimize the pathway in a multi-level modular manner^17^. These engineered cyanobacterial strains produced 29.9 mg/L^15^, 404 mg/L^16^, and 4.8 g/L^17^ of *n*-butanol. However, the low energy conversion efficiency (<3%) of natural photosynthesis^18^ makes the use of cyanobacteria not ideal for *n*-butanol production.

To enhance energy conversion efficiency for biofuel production, artificial photosynthesis, where photo-generated electrons were used to drive chemical reactions^19^, was developed. However, due to catalyst limitations, hydrogen (H_2_) was produced as the main product^20^. Although H_2_ can be used as a fuel, using such an explosive gas requires significant modifications to the current gasoline-based infrastructures^21,22^. To avoid this, an H_2_-consuming chemoautotrophic bacterium *Ralstonia eutropha* was used for producing carbon-based liquid fuels using a hybrid water-splitting biosynthetic system. In this system, H_2_ and O_2_ were produced from water splitting (powered by electricity from a potentiostat) using a cobalt phosphorus catalyst with an applied electrical potential (*E*_appl_) of 2.0 V^19^. The H_2_ was then fed to the engineered *R. eutropha* to synthesize C_3_–C_5_ alcohol or polyhydroxybutyrate (PHB) from CO_2_^19^. This hybrid biosynthetic system reached an electrical energy conversion efficiency (EECE) up to ~20% using air (consists of 400 ppm CO_2_) toward biomass^19^. These values far exceeded the energy conversion efficiency of natural photosynthesis. Also, using genetically modified *R. eutropha*, the system reached an EECE of 16 ± 2% towards C_4_-C_5_ alcohol using pure CO_2_. Also, coupling a solar panel with biosynthetic system resulted in an energy conversion efficiency of 6% towards biomass using pure CO_2_^23^. These studies indeed provided a platform for indirect solar fuel production from CO_2_. However, this technology may not be an efficient and economical method for biofuel synthesis because, (1) it produces O_2,_ which is detrimental to many biofuel synthesis processes;^14^ (2) it uses H_2_ as an electron donor, which due to its low solubility limits electron transfer efficiency^24^ and, finally; (3) this system requires electrical potentials higher than 1.23 V^18^, making it an expensive method on the market. Therefore, it is critical to look for organisms that can overcome these limitations to advance carbon-neutral biofuel production.

One such organism is the anoxygenic photoautotroph *Rhodopseudomonas palustris* TIE-1 (TIE-1). TIE-1 can use various carbon sources, such as atmospheric CO_2_ and organic acids that can be easily obtained from organic wastes^25^. TIE-1 can also fix dinitrogen gas (N_2_)^26^ and use a wide range of electron sources. These include H_2,_ which is a byproduct of many industries; ferrous iron [Fe(II)], which is a naturally abundant element^27,28^. Most importantly, TIE-1 can also use electrons from poised electrodes (i.e., photoelectroautotrophy), that can be generated sustainably, for its photosynthetic growth^27,29–32^. This wide electron donor selection enables TIE-1 to perform photosynthesis while avoiding O_2_ generation, a harmful component for biofuel synthesis^14^. TIE-1’s ability to perform photoelectroautotrophy is advantageous for biofuel production, because the direct electron uptake by TIE-1 from a poised electrode avoids the need of an indirect electron donor such as H_2_. TIE-1 has a low E_appl_ (0.1 V)^27,29–32^ requirement, which lowers cost and electrochemical O_2_ generation. TIE-1’s *E*_appl_ requirement is ~90% lower than that needed for water-splitting^19^, which allows the use of low-cost solar panels to build novel biohybrid systems for solar fuel synthesis. Overall, TIE-1 is a superlative biocatalyst that allows us to use extant CO_2_, N_2_, solar energy, and electrons generated by renewable electricity for bioproduction. This process enables excess electricity to be stored as a usable fuel or product for later use.

In a previous study, *R. palustris* CGA009 (CGA009), a strain closely related to TIE-1, was engineered to produce *n*-butanol from *n*-butyrate^33^. In that study, the gene encoding the alcohol/aldehyde dehydrogenases (AdhE2) from *R. palustris* Bisb18 was codon-optimized and introduced into CGA009^33^. When cultured in the absence of CO_2_, the modified CGA009 was forced to reduce *n*-butyrate into *n*-butanol to maintain the redox balance^33^. Although this study used a phototroph for *n*-butanol production, the use of an organic substrate makes this approach carbon-positive.

To produce *n*-butanol in a sustainable and carbon-neutral manner, we introduced an efficient, codon-optimized *n*-butanol biosynthesis pathway into TIE-1. This pathway was assembled using irreversible and efficient enzymes and produced 4.6 g/L *n*-butanol in *E. coli*^34^. The pathway contains five genes (*phaA, phaB, phaJ, ter, adhE*2)^27^. Because TIE-1 possesses homologs for the first two genes (*phaA* and *phaB*)^27^, we designed two different cassettes (Fig. 1b), containing either the whole (5-gene cassette) or a partial *n*-butanol biosynthesis pathway (3-gene cassette). As shown in Fig. 1a, carbon (acetyl-CoA) and reducing equivalents (NADH) are two major substrates for *n*-butanol biosynthesis. Previous studies in cyanobacteria have shown that a PHB synthase deletion mutant produces more butanol^35^, and a glycogen synthase deletion mutant showed higher carbon conversion efficiency (CCE) towards iso-butanol^35,36^. We, therefore, constructed TIE-1 knockout mutants lacking hydroxybutyrate polymerase (*phaC*1, Rpal_2780 and *phaC*2, Rpal_4722) or glycogen synthase (*glgA*, Rpal_0386). As expected, these mutants accumulated significantly less PHB and glycogen compared to wild-type (WT) (Supplementary Fig. 1). The detailed carbon flow between *n*-butanol synthesis, PHB synthesis, and glycogen synthesis is shown in Supplementary Fig. 2. Previous studies suggested that nitrogenase deletion mutants possess a more reduced intracellular environment in *Rhodobacter capsulatus* and CGA009^37–39^. We predicted this would be true in TIE-1 as well and created a double mutant (Nif mutant) by deleting *nifA1* (Rpal_5113) and *nifA2* (Rpal_1624), which are the regulators that potentially activate their cognate clusters of nitrogenase genes^40^, namely the putative Molybdenum-dependent nitrogenase and the putative Iron-dependent nitrogenase^40^. The increased NADH/NAD^+^ ratio observed in the Nif mutant compared to WT, when using 3-hydroxybutyrate as electron donor (Supplementary Fig. 3) indicated a more reduced intracellular environment. In addition, the inability of the Nif mutants to grow under nitrogen-fixing conditions (Supplementary Table 1) further indicated the loss of nitrogen-fixing capability of the Nif mutant. After introducing the 3-gene cassette/5-gene cassette into the TIE-1 WT and mutant strains, we tested *n*-butanol production under both photoheterotrophic (Fig. 1c) and photoautotrophic (Fig. 1d) conditions. Under photoelectroautotrophy, we used a novel hybrid bioelectrochemical cell (BEC) platform powered by electricity supplied from either potentiostat or a solar panel.

Our results show that the anoxygenic phototroph TIE-1 can produce *n*-butanol sustainably using organic acids or CO_2_ as a carbon source, light as an energy source, and H_2_, Fe(II), or electrons from renewably generated electricity as an electron source. To the best of our knowledge, this study represents the first attempt for biofuel production using a solar panel-powered microbial electrosynthesis platform, where CO_2_ is directly converted to liquid fuel. Overall, these results show that TIE-1 can be an attractive future microbial chassis for producing carbon-neutral biofuels via synthetic biology and metabolic engineering, building upon our work using WT TIE-1 for bioplastic production^27^.

## Results

### Deleting an electron-consuming pathway or low cell growth enhances *n*-butanol production

We measured *n*-butanol production by WT with 3-gene cassette (WT-3), WT with 5-gene cassette (WT-5), and TIE-1 mutants with either 3-gene (Nif-3, Gly-3, Phb-3) or 5-gene cassette (Nif-5, Gly-5), under various photoheterotrophic and photoautotrophic conditions (substrate combinations, incubation time and final optical density listed in Supplementary Tables 2, 3 and 4) to identify the most productive strains and conditions. Because the adaptation phase that we performed for slow growth conditions such as nitrogen-fixing conditions resulted in plasmid loss, we performed all the experiments by incubating dense aerobically pre-grown cells (OD_660_ = 1). These cells were washed and used as inoculum for the anaerobic conditions using various electron and carbon sources. The Nif mutants incubated under nitrogen-fixing conditions are non-growing. For all the other mutants and incubation conditions, the final optical density is listed in Supplementary Table 4.

For photoheterotrophic conditions, we chose acetate (Ac) or 3-hydroxybutyrate (3Hy) as carbon and electron sources because both substrates enter the *n*-butanol biosynthesis pathway directly as their CoA derivatives (Supplementary Fig. 4)^41^. For photoautotrophic conditions, we used either H_2_ or Fe(II) as an electron donor. CO_2_ was supplied in all conditions to maintain the pH of the medium and for redox balance in the cell. We provided N_2_ or ammonia (NH_4_^+^) as the nitrogen source.

**Fig. 2:**
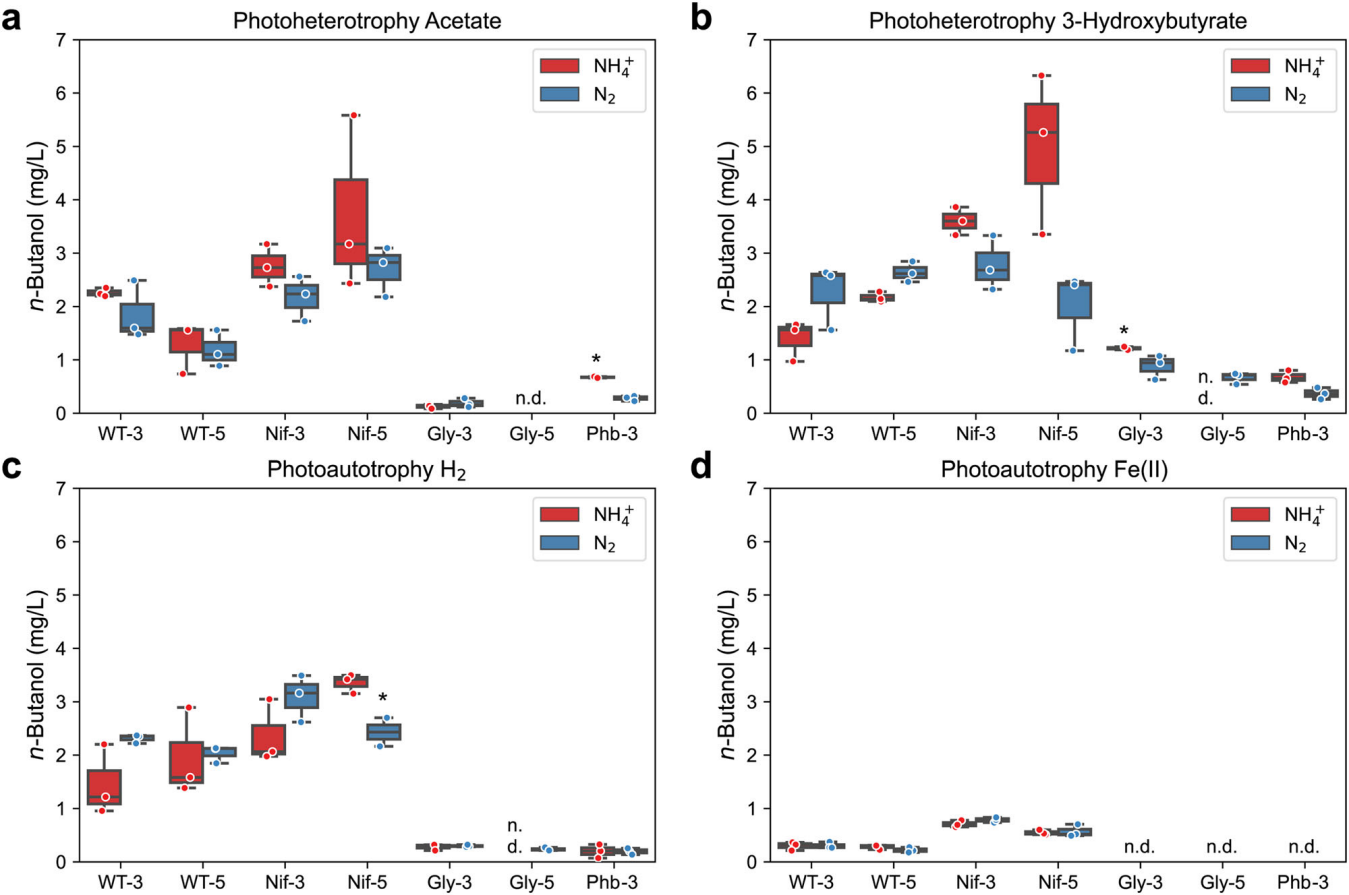
The nitrogenase double mutant (Nif) produced the highest amount of *n*-butanol in the presence of 3-hydroxybutyrate. The concentration of *n*-butanol in mg/L when TIE-1 was cultured with ammonium (NH_4_^+^, red) or dinitrogen gas (N_2_, blue) and **a** acetate (photoheterotrophy) **b** 3-hydroxybutyrate (photoheterotrophy) **c** hydrogen (H_2_) (photoautotrophy) and **d** ferrous iron [Fe(II)] (photoautotrophy). CO_2_ was present in all conditions. Data are from *n* = 3 of independent experiments. Boxes that only have two biological replicates are indicated by ‘*‘. WT-3: wild type with 3-gene cassette; WT-5: wild type with 5-gene cassette; Nif- 3: nitrogenase knockout t with 3-gene cassette; Nif - 5: nitrogenase knockout with 5-gene cassette; Gly- 3: glycogen synthase knockout with 3-gene cassette; Gly- 5: glycogen synthase knockout with 5-gene cassette; Phb- 3: hydroxybutyrate polymerase knockout with 3-gene cassette, n.d. (non-detectable).

We found that depending on the carbon and electron source, the same construct produced variable amounts of *n*-butanol. *n*-Butanol production was the highest in the presence of 3Hy, followed by H_2_, Ac, and Fe(II) (Fig. 2). We found that Nif-5 is the most efficient *n*-butanol producer with the highest production of 4.98 ± 0.87 mg/L under the photoheterotrophic conditions with NH_4_^+^ (Fig. 2b). The same construct, however, produced ~10-fold lower *n*-butanol when incubated with Fe(II) (0.55 ± 0.03 mg/L) (Fig. 2d).

Compared to WT-3/WT-5, Nif-3/Nif-5 produced similar or more *n*-butanol depending on the substrates, whereas Gly-3/Gly-5 and Phb-3 produced less *n*-butanol regardless of the substrate type (Fig. 2). The presence of NH_4_^+^ in the media has been reported to repress the nitrogenase genes^42^. Therefore, we speculated that in its presence, WT-3/WT-5 and Nif-3/Nif-5 would produce similar amounts of *n*-butanol. Surprisingly, in most cases, Nif-3/Nif-5 produced a higher amount of *n*-butanol than the WT-3/WT-5, even in the presence of NH_4_^+^ (Fig. 2). Overall, we observe that deleting an electron-consuming pathway (Nif) is beneficial, whereas deleting an acetyl-CoA-consuming pathway (Gly and Phb) is detrimental to *n*-butanol production. However, the increased *n*-butanol production in Nif-3/Nif-5 could also be a result of low cell growth (Supplementary Table 4).

**Fig. 3:**
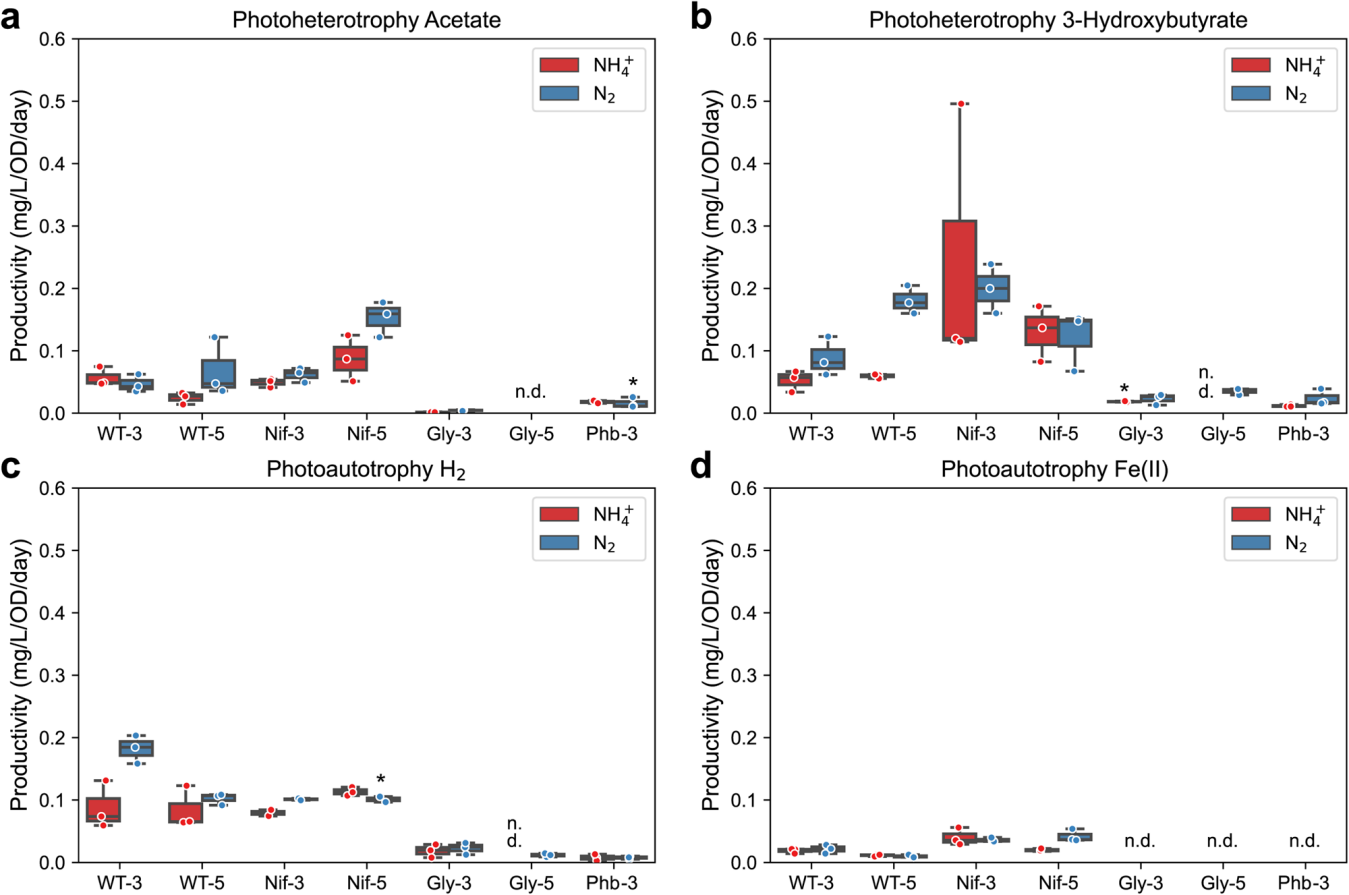
3-Hydroxybutyrate resulted in the highest *n*-butanol productivity. The *n*-butanol productivity when TIE-1 was cultured with ammonium (NH_4_^+^, red) or dinitrogen gas (N_2_, blue) and **a** acetate (photoheterotrophy) **b** 3-hydroxybutyrate (photoheterotrophy) **c** hydrogen (H_2_) (photoautotrophy) and **d** ferrous iron [Fe(II)] (photoautotrophy). CO_2_ was present in all conditions. Data are from *n* = 3 of independent experiments. Boxes with data from *n* = 2 independent experiments are indicated by ‘*’. WT-3: wild type with 3-gene cassette; WT-5: wild type with 5-gene cassette; Nif-3: nitrogenase knockout t with 3-gene cassette; Nif-5: nitrogenase knockout with 5-gene cassette; Gly-3: glycogen synthase knockout with 3-gene cassette; Gly-5: glycogen synthase knockout with 5-gene cassette; Phb-3: hydroxybutyrate polymerase knockout with 3-gene cassette, n.d. (non-detectable).

Because the multiple electron donor choices resulted in different incubation times, and final optical density, we calculated the productivity (*n*-butanol mg/L/day/OD_660_) by each strain under each incubation condition to make a meaningful comparison. For most strain constructs, incubation with 3Hy showed the highest productivity, followed by H_2_, Ac, and Fe(II) (Fig. 3a–d). Among all the strain constructs, Nif-3/Nif-5 showed the highest productivity under most incubation conditions.

No *n*-butanol was detected from WT with an empty vector using 3Hy as a carbon source and NH_4_^+^ as a nitrogen source. To ensure the *n*-butanol production is not toxic to TIE-1^7^, we performed a toxicity assay. The lowest inhibitory concentration of *n*-butanol for TIE-1 is 4050 mg/L (Supplementary Table 5), which is about 1000-fold higher than the highest *n*-butanol produced in our experiment (4.98 ± 0.87 mg/L). Hence, the *n*-butanol produced during our study does not limit the growth of TIE-1.

### Deleting acetyl-CoA-consuming pathways diverts carbon to acetone production

**Fig. 4:**
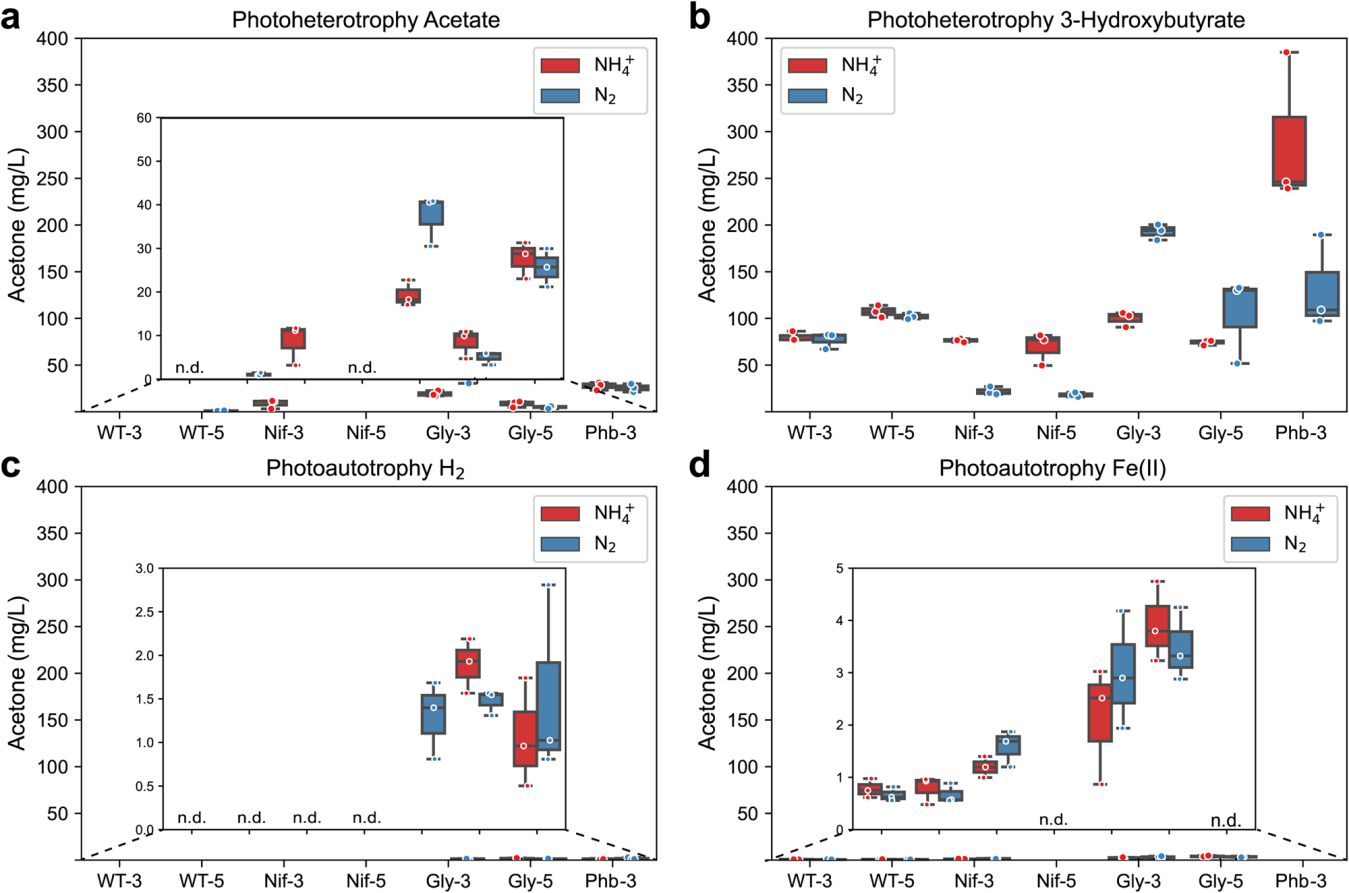
High *n*-butanol production correlates to low acetone production amongst TIE-1 mutants. The concentration of acetone in mg/L when TIE-1 was cultured with ammonium (NH_4_^+^, red) or dinitrogen gas (N_2_, blue) and **a** acetate (photoheterotrophy) **b** 3-hydroxybutyrate (photoheterotrophy) **c** hydrogen (H_2_) (photoautotrophy) and **d** ferrous iron [Fe(II)] (photoautotrophy). CO_2_ was present in all conditions. Data are from *n* = 3 of independent experiments. WT-3: wild type with 3-gene cassette; WT-5: wild type with 5-gene cassette; Nif-3: nitrogenase knockout with 3-gene cassette; Nif-5: nitrogenase knockout with 5-gene cassette; Gly-3: glycogen synthase knockout with 3-gene cassette; Gly-5: glycogen synthase knockout with 5-gene cassette; Phb-3: hydroxybutyrate polymerase knockout with 3-gene cassette, n.d. (non-detectable).

Acetone is a major byproduct of *n*-butanol biosynthesis^41^ which is produced by the accumulation of acetoacetyl-CoA, an intermediate product in *n*-butanol biosynthesis^7,41^ (Fig. 1a and Supplementary Fig. 4). We observed the highest acetone production by Phb-3 (0.00 to 290.01 ± 47.51 mg/L) followed by Gly-3/Gly-5 (1.47 ± 0.08 mg/L to 192.84 ± 4.82 mg/L), WT-3/WT-5 (0.00 mg/L to 107.39 ± 3.74 mg/L), and Nif-3/Nif-5 (0.00 mg/L to 76.44 ± 1.12 mg/L) (Fig. 4). This acetone production trend is in the reverse trend of *n*-butanol production, i.e., Nif-3/Nif-5 produced the highest, and the Phb-3 produced the lowest amount of *n*-butanol (Fig. 2). These results indicate that acetone biosynthesis likely competes for acetyl-CoA with *n*-butanol biosynthesis. Using either Phb or Gly, acetyl-CoA that would have otherwise been directed toward PHB or glycogen synthesis was diverted to acetone biosynthesis.

Compared to using Ac as a substrate, which produced 0.00 to 37.29 ± 3.40 mg/L of acetone, all constructs produced ~10-100-fold more acetone when supplied with 3Hy (18.15 ± 1.41 to 290.10 ± 38.80 mg/L (Fig. 4a, b). However, when the same strain was used, the acetone production under photoautotrophic conditions was lowered by ~25–125-fold compared to photoheterotrophic conditions with only 0.00 to 3.92 ± 0.44 mg/L (Fig. 4c, d). These results indicate that under photoheterotrophic conditions, particularly with 3Hy, TIE-1 accumulates more acetyl-CoA, which is eventually converted into acetone. The high acetone production suggests that acetyl-CoA is not limiting *n*-butanol production. We also tested acetone toxicity in TIE-1 and found that the amount of acetone produced does not limit TIE-1’s growth (Fig. 4, Supplementary Table 6).

### More reducing equivalents or low cell growth enhances carbon conversion efficiency (CCE) to *n*-butanol

**Fig. 5:**
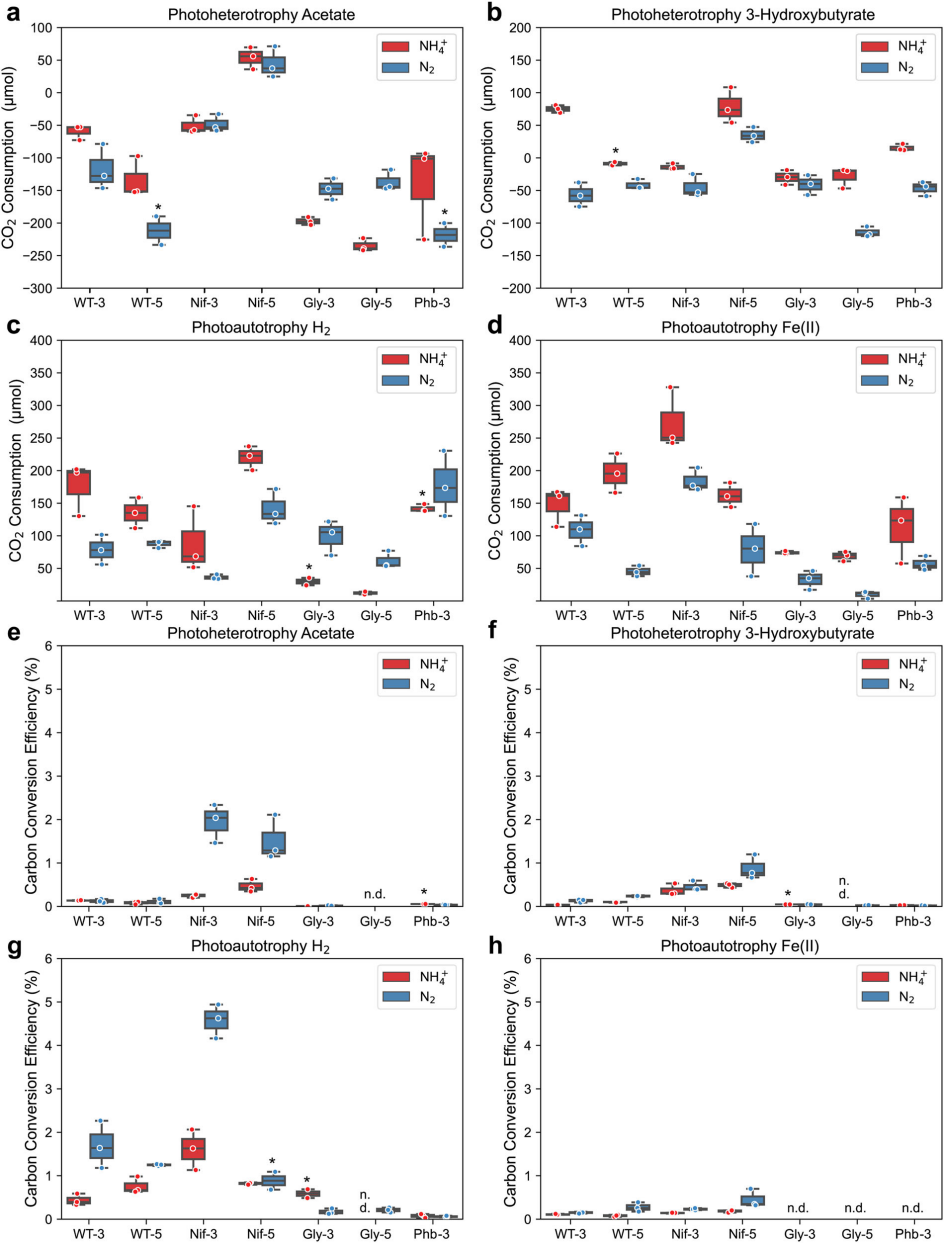
The nitrogenase double mutant (Nif) converts carbon to *n*-butanol more efficiently. **a**–**d** The CO_2_ consumption (positive value)/production (negative value). **e**–**h** Carbon conversion efficiency to *n*-butanol. **a**, **e**: acetate (photoheterotrophy) **b**, **f**: 3-hydroxybutyrate (photoheterotrophy) **c**, **g**: hydrogen (H_2_) (photoautotrophy) and **d**, **h**: ferrous iron [Fe(II)] (photoautotrophy). CO_2_ was present in all conditions. Data are from *n* = 3 of independent experiments. WT-3: wild type with 3-gene cassette; WT-5: wild type with 5-gene cassette; Nif-3: nitrogenase knockout with 3-gene cassette; Nif-5: nitrogenase knockout with 5-gene cassette; Gly-3: glycogen synthase knockout with 3-gene cassette; Gly-5: glycogen synthase knockout with 5-gene cassette; Phb-3: hydroxybutyrate polymerase knockout with 3-gene cassette, n.d. non-detectable.

To further identify the most efficient strain and substrate for *n*-butanol production with respect to carbon, we determined carbon consumption (Supplementary Fig. 5a, b, and Fig. 5a–d and CCE (Fig. 5e–h) towards *n*-butanol for each construct under all conditions.

#### Carbon consumption

We have recently shown that TIE-1 can fix CO_2_ during photoheterotrophic growth^30,42^. Therefore, we also calculated CO_2_ consumption and generation by all constructs. Under photoheterotrophy, all TIE-1 constructs consumed more (or generated less, represented by smaller negative value) CO_2_ with 3Hy (up to −114.23 ± 4.52 to 78.67 ± 15.86 μmol) than Ac (up to –243.67 ± 5.79 to 53.79 ± 9.77 μmol) (Fig. 5a, b). With either 3Hy or Ac, Nif-3/Nif-5 consumed more CO_2_ (or generated less) (CO_2_ generation: −50.53 ± 8.01 to 78.67 ± 15.86 μmol) (Fig. 5a, b). These results are consistent with a previous study where the use of a more reduced substrate (such as 3Hy) resulted in more carbon consumption than the use of a more oxidized substrate (such as acetate) for redox balance^42^.

Similarly, under photoautotrophic conditions, Nif-3/Nif-5 consumed the highest amount of CO_2_ (36.41 ± 2.17 to 273.76 ± 27.25 μmol), except for Nif-3 incubated with H_2_ and NH_4_^+^ (Fig. 5c, d). This observation is likely due to the higher CO_2_ fixation required to achieve redox balance in the absence of N_2_-fixation. Gly-3/Gly-5 consumed the lowest amount of CO_2_, ranging from −234.67 ± 5.79 to 99.04 ± 15.32 μmol (Fig. 5c, d). A previous study using glycogen mutants has been reported to fix less CO_2_ compared to WT in cyanobacteria^36^. This observation corroborates with our finding that Gly-3/Gly-5 produces low *n*-butanol under photoautotrophic conditions (Fig. 2c, d).

#### CCE

CCE is defined as moles of carbon in *n*-butanol divided by moles of carbon from substrates. (see method section for detailed calculations) Similar to the trend for *n*-butanol production (Fig. 2), Nif-3/Nif-5 showed the highest CCE towards *n*-butanol (0.12 ± 0.03 to 4.58 ± 0.23%), followed by WT-3/WT-5 (0.03 ± 0.01 to 1.70 ± 0.32%), Gly-3/Gly-5 (0.00 to 0.59 ± 0.10%), and Phb-3 (0.00 to 0.16 ± 0.04%) (Fig. 5e–h). These results suggest that excess reducing equivalents enhanced *n*-butanol production and facilitated CCE to *n*-butanol. Similar to the results from *n*-butanol production, this could also be due to low cell growth. In contrast, lack of the PHB or glycogen biosynthesis decreased overall CCE to *n*-butanol. We found that all strains had the highest CCE when incubated with H_2_ (0.00 to 4.58 ± 0.23%), except for Phb (Fig. 5g), which was unable to produce *n*-butanol using any substrate (Fig. 2c). This high CCE in the presence of H_2_ (Fig. 5g) could be due to low acetone production (Fig. 4c) and the lower cell growth compared to the other conditions.

Higher CCE towards *n*-butanol (1- to 7-fold) was observed when Nif-3/Nif-5 was supplied with N_2_ compared to NH_4_^+^. For example, in the presence of NH_4_^+^, Nif-3/Nif-5 showed CCE of 0.14 ± 0.01 to 1.61 ± 0.27%, which increased to 0.23 ± 0.01 to 4.58 ± 0.23% when N_2_ was provided (Fig. 5e–h). This result also indicated that low cell growth might lead to higher CCE towards *n*-butanol biosynthesis by TIE-1.

### More reducing equivalents and low cell growth enhances electron conversion efficiency to *n*-butanol

**Fig. 6:**
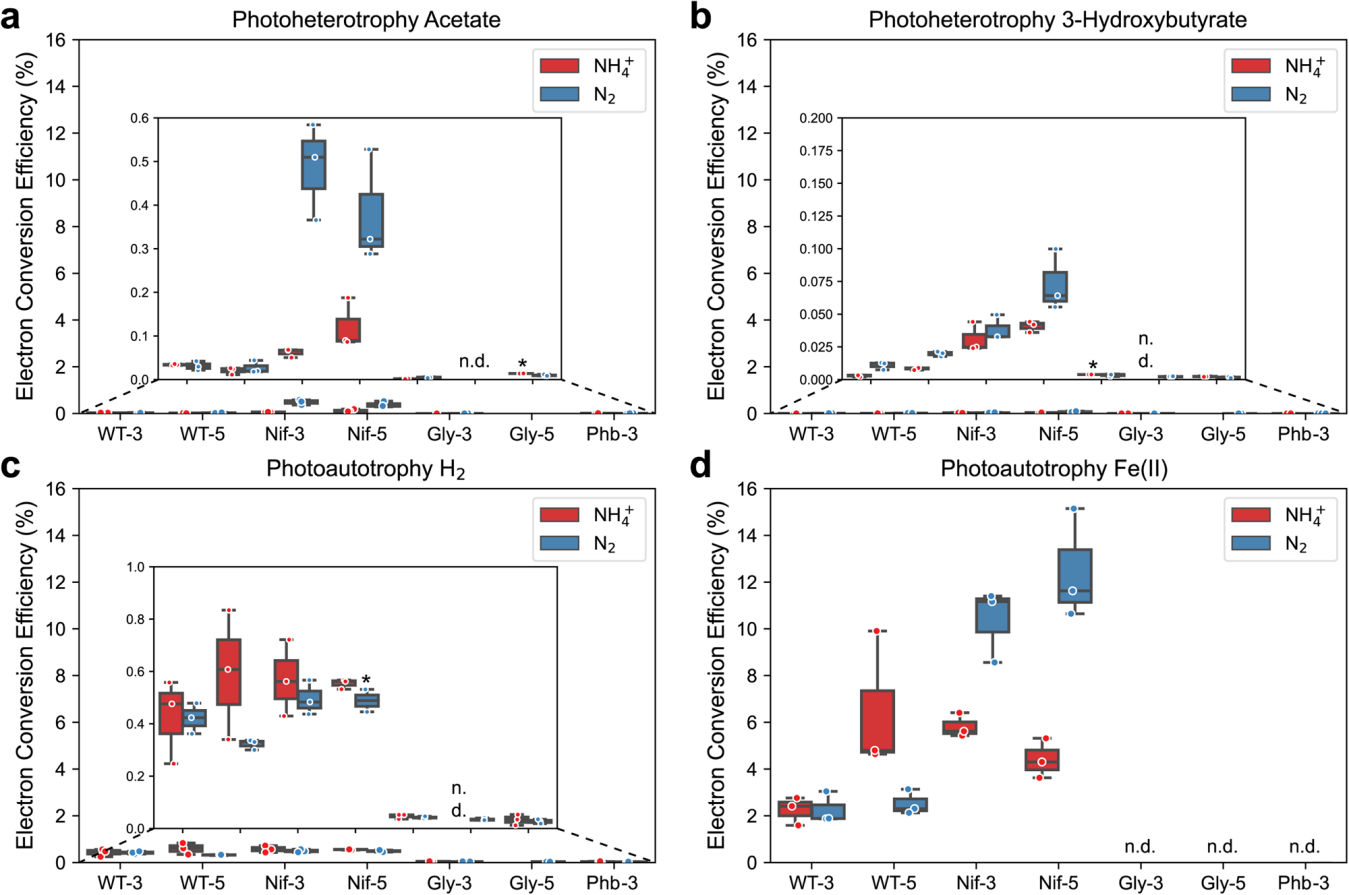
The nitrogenase double mutant (Nif) converts electrons to *n*-butanol more efficiently. The electron conversion efficiency towards *n*-butanol (%) when TIE-1 was cultured with ammonium (NH_4_^+^, red) or dinitrogen gas (N_2_, blue) and **a** acetate (photoheterotrophy) **b** (3-hydroxybutyrate (photoheterotrophy) **c** hydrogen (H_2_) (photoautotrophy); and **d** ferrous iron [Fe(II)] (photoautotrophy). CO_2_ was present in all conditions. Data are from *n* = 3 of independent experiments. Boxes that only have two biological replicates are indicated by ‘*’. WT-3: wild type with 3-gene cassette; WT-5: wild type with 5-gene cassette; Nif-3: nitrogenase knockout t with 3-gene cassette; Nif-5: nitrogenase knockout with 5-gene cassette; Gly-3: glycogen synthase knockout with 3-gene cassette; Gly-5: glycogen synthase knockout with 5-gene cassette; Phb-3: hydroxybutyrate polymerase knockout with 3-gene cassette, n.d. (non-detectable).

To further identify the most productive strain and substrate toward *n*-butanol production with respect to electron availability, we calculated each construct’s electron conversion efficiency (ECE) towards *n*-butanol (electron donor consumption data shown in Supplementary Fig. 5). ECE is defined as the moles of the electron in *n*-butanol divided by the moles of the electron from substrates (see method section for detailed calculations). We found that photoautotrophic conditions reached higher ECE towards *n*-butanol than photoheterotrophic conditions. With an ECE of 0.00 to 12.47 ± 1.37%, Fe(II) was the most favorable electron donor followed by H_2_ (0.00 to 0.59 ± 0.14%), Ac (0.00 to 0.49 ± 0.06%), and 3Hy (0.00 to 0.07 ± 0.01%) (Fig. 6a–d). The highest ECE towards bioplastic in the presence of Fe(II) has also been observed previously^27^.

**Fig. 7:**
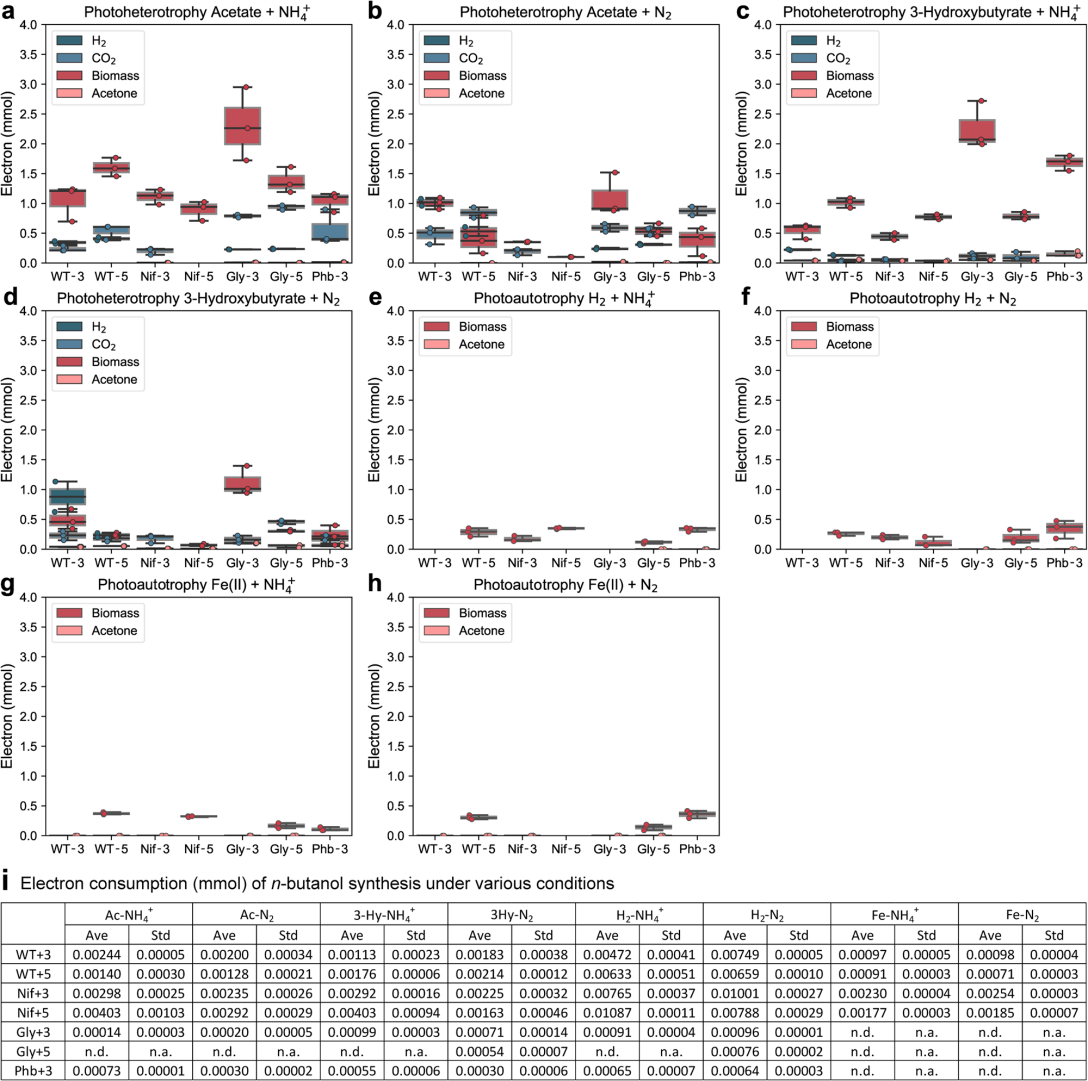
Using Hydrogen as an electron donor resulted in low electron flow in byproduct generation. Electron consumption of *n*-butanol/acetone/H_2_/CO_2_/biomass synthesis under various incubation conditions. **a** acetate + NH_4_^+^ (photoheterotrophy) **b** acetate + N_2_ (photoheterotrophy) **c** 3-hydroxybutyrate + NH_4_^+^ (photoheterotrophy) **d** 3-hydroxybutyrate  + N_2_ (photoheterotrophy) **e** hydrogen (H_2_) + NH_4_^+^ (photoautotrophy) **f** hydrogen (H_2_) + N_2_ (photoautotrophy) **g** ferrous iron [Fe(II)] + NH_4_^+^ (photoautotrophy) **h** ferrous iron [Fe(II)] + N_2_ (photoautotrophy) **i** electron consumption of *n*-butanol synthesis under various incubation conditions.CO_2_ was present in all conditions. Data are from *n* = 3 of independent experiments. WT-3: wild type with 3-gene cassette; WT-5: wild type with 5-gene cassette; Nif-3: nitrogenase knockout t with 3-gene cassette; Nif-5: nitrogenase knockout with 5-gene cassette; Gly-3: glycogen synthase knockout with 3-gene cassette; Gly-5: glycogen synthase knockout with 5-gene cassette; Phb-3: hydroxybutyrate polymerase knockout with 3-gene cassette n.d. is non-detectable and n.a. is not available.

To better understand the distributions of electrons in acetone/CO_2_/H_2_/biomass synthesis, we calculated the electrons consumed by *n*-butanol biosynthesis, acetone biosynthesis, biomass, CO_2_ generation, and H_2_ generation. For all incubation conditions, biomass is the most significant electron sink (Fig. 7). However, under heterotrophic conditions (Fig. 7a–d), CO_2_ and H_2_ generation also act as major electron sinks for most of the strains. The electron consumed by acetone/*n*-butanol biosynthesis is relatively insignificant compared to biomass, CO_2,_ and H_2_ generation. Under autotrophic conditions (Fig. 7e–h), CO_2_ was not generated but consumed, hence we did not consider the electron consumption in generating CO_2_. Similarly, when H_2_ was used as an electron donor, H_2_ was consumed, consequently, we did not consider the electron consumption in generating H_2_ either. Furthermore, no H_2_ generation was observed when Fe(II) was used as the electron donor.

Using the same carbon and electron source, the highest ECE was achieved by Nif-3/Nif-5 (0.04 ± 0.01 to 12.47 ± 1.37%), followed by WT-3/WT-5 (0.00 to 6.45 ± 1.73%), Gly-3/Gly-5 (0.00 to 0.05 ± 0.00%), and Phb + 3 (0.00 to 0.03 ± 0.01%, Figs. 6a–d). In summary, the availability of reducing equivalents due to deletion of an electron-consuming pathway and low cell growth (Nif-3/Nif-5) leads to higher ECE for *n*-butanol biosynthesis in TIE-1.

### *n*-Butanol bioproduction can be achieved with light, electricity, and CO_2_

**Fig. 8:**
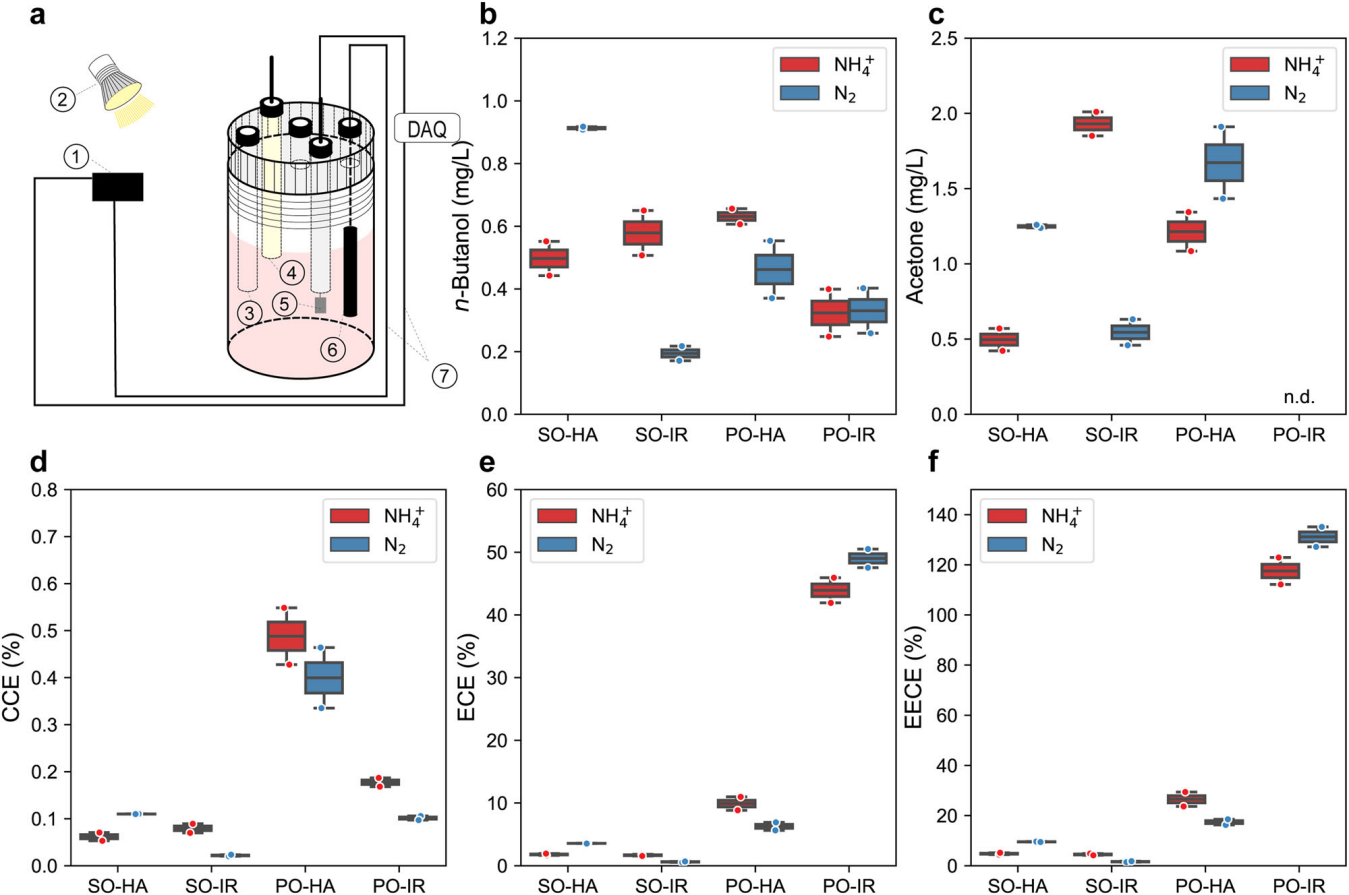
**Three-electrodes configured sealed type bioelectrochemical cell (BEC),** *n*-butanol production, acetone production, carbon conversion efficiency, electron conversion efficiency, and electrical energy conversion efficiency (EECE) towards *n*-butanol by the nitrogenase double mutant with the 5-gene cassette under photoelectroautotrophy. Under photoelectroautotrophic conditions, TIE-1 gains electrons from a poised electrode, using light as an energy source and carbon dioxide as a carbon source. For all the platforms, either ammonium (NH_4_^+^) or dinitrogen gas (N_2_) was supplied. **a** Schematic set up of BEC platform. platform set up: 1- electricity source 2-light source, 3- Purge inlet, 4- Reference electrode (Ag/AgCl in 3 M KCl), 5- Counter electrode (Pt foil, 5 cm^2^), 6- Working electrode (Graphite rod, 3.2 cm^2^), 7- electrical wire DAQ- Data acquisition); **b** *n*-butanol production; **c** acetone production; **d** carbon conversion efficiency (CCE) towards *n*-butanol; **e** electron conversion efficiency (ECE) towards *n*-butanol; **f** electrical energy conversion efficiency (EECE) towards *n*-butanol. PO potentiostat, IR infrared light, HA halogen light, SO solar panel. Data are from *n* = 2 of independent experiments.

We have recently demonstrated that the photoelectroautotrophic growth of TIE-1 leads to a highly reduced intracellular environment compared to other growth conditions^30^. Under photoelectroautotrophy, TIE-1 can attach to the poised electrode to form a biofilm, and can gain electrons via direct extracellular electron uptake^30^. This process is performed by a complex formed by a single periplasmic decaheme cytochrome *c*, PioA, an outer membrane porin, PioB, that allows electron transfer across the outer membrane, and PioC a periplasmic electron shuttle^43^. We investigated *n*-butanol production by TIE-1 under photoelectroautotrophy using a three-electrode sealed BEC (Fig. 8a). For this experiment, we used Nif-5 as it was the most efficient *n*-butanol producer under most of the tested conditions (Figs. 2c, 5c, 6g).

We created four distinct biofuel production BEC platforms by combining two different electricity sources (grid-powered potentiostat or a solar panel) with two light sources (infrared or halogen light). In the case of BECs powered by potentiostat, the bioreactors use a three-electrodes system, wherein a reference electrode was used to control the poised potential towards the working electrode steadily at *E*_appl_ 0.5 V. In contrast, for the BECs powered by solar panel, the bioreactors use a two-electrode system, wherein the poised potential was controlled by the existing voltage of the solar panel (*E*_appl_ 0.5 V) from its positive and negative terminals (like voltage obtained in a battery). The different electrode configuration and control modes could lead to significant discrepancies in the reactor performance. For example, using the abiotic control, the BECs powered by solar panels resulted in nearly 25-fold higher electron uptake than the BEC powered by the potentiostat (Supplementary Fig. 6a). Further, the potentiostat approach represents conventional electrical sources, while the solar panel approach allows us to leverage renewably generated electricity. Infrared light is only a small portion of the solar spectrum that specifically excites the photosystem of TIE-1^26^. Halogen light mimics natural sunlight that represents the solar spectrum^44,45^. So, it can excite the photosystem of TIE-1 and support electricity generation by a solar panel simultaneously. *BEC platform 1* used solar panel generated electrons and halogen light; *BEC platform 2* used solar panel generated electrons and infrared light; *BEC platform 3* used potentiostat and halogen light; *BEC platform 4* used potentiostat and infrared light. Either N_2_ or NH_4_^+^ was supplied as the nitrogen source. Supplementary Table 7 lists detailed platform setups. We measured *n*-butanol production, acetone production and calculated CCE and ECE (measured as coulombic efficiency) towards *n*-butanol for each platform. We also calculated the EECE towards *n*-butanol by dividing the combustion heat of the produced *n*-butanol by the electrical energy input.

The highest (0.91 ± 0.07 mg/L) and the lowest (0.19 ± 0.02 mg/L) *n*-butanol production was achieved when N_2_ was supplied as a nitrogen source in *BEC platform 1* and *BEC platform 2*, respectively (Fig. 8b). The BEC platforms powered by solar panels showed 3–8 -fold higher CO_2_ consumption (Supplementary Fig. 6b) and 5 to 40 -fold higher electron uptake (Supplementary Fig. 6c)^30,42^ compared to the BEC platforms powered by the grid-powered potentiostat. Similar to the other autotrophic conditions (Fig. 4c, d), little or no acetone was produced (Fig. 8c) from the BEC platforms. *BEC platform 4* achieved the highest CCE towards *n*-butanol (0.49 ± 0.06%, Fig. 8d) compared to the other three BEC platforms. Although BEC platforms powered by grid-powered potentiostat achieved much lower electron uptake (Supplementary Fig. 6c), they reached a much higher ECE (6–25-folds) than the platforms powered by a solar panel (Fig. 8e).

BEC platforms under halogen light achieved higher ECE (4 to 8-fold, except using solar panel incubated with NH_4_^+^, Fig. 8e) compared to the BEC platforms using infrared light. However, the BEC platforms illuminated by halogen light (platforms 1 and 3) had much lower (20– 90%) electron uptake, particularly when using solar panels as an electricity source (Supplementary Fig. 6c). To ensure that a lower number of attached cells did not reduce electron uptake from the platforms using halogen light, we performed a live-dead viability assay. We observed that the percentage of live cells attached to the electrodes was similar in all the BEC platforms (40–50%) (Supplementary Fig. 6d, e). This indicates that halogen light is not the ideal light source for TIE-1 with respect to electron uptake.

We further compared the EECE towards *n*-butanol between the two electricity sources. We found that the BEC platforms powered by solar panel show lower EECE (1.62 ± 0.20 to 9.55 ± 0.34%) than the BEC platforms using a potentiostat (16.62 ± 1.01 to 131.13 ± 3.97%) when the same nitrogen source (either N_2_ or NH_4_) was supplied (Fig. 8f). With respect to the light source, platforms using halogen light resulted in higher EECE (4.80 ± 0.38 to 131.14 ± 3.97%) than platforms using infrared light (1.62 ± 0.20 to 26.52 ± 2.87%) when the same nitrogen source was supplied (Fig. 8f). Halogen light represents the solar spectrum, and several wavelengths from this light source can be absorbed by TIE-1 via the light-harvesting complexes and, eventually, the photosystem^30,46^. This would lead to higher ATP synthesis via cyclic photosynthesis by TIE-1^30^, perhaps explaining the >100% EECE.

We also calculated the energy conversion efficiency (η_totalc_) towards *n*-butanol from light for all four systems (Supplementary Fig. 7). Comparing η_totalc_ between the two electricity sources (i.e., platforms 1 and 2 vs. 3 and 4), when supplied with the same nitrogen source (either N_2_ or NH_4_^+^), platforms 2 reached lower η_totalc_ than platforms 4 (Supplementary Fig. 7). No significant difference was noticed for η_totalc_ between platforms 1 and 3 when incubated with NH_4_^+^. However, when incubated with N_2_, platform 1 had higher η_totalc_ compared to platform 3. As for the light sources, in general, when the same nitrogen source was used, halogen light resulted in lower η_totalc_ than infrared light [except using solar panel as an electricity source and incubated with N_2_ (Supplementary Fig. 7)]. These results suggest that TIE-1 prefers infrared light over halogen light^26,47^.

In summary, *BEC platform 1* showed higher *n*-butanol production, *BEC platform 4* showed the highest CCE towards *n*-butanol, and *BEC platform 3* showed the ECE and EECE towards *n*-butanol. Although *BEC platform 1* resulted in moderate conversion efficiencies, the highest *n*-butanol production (up to fivefold) with the use of sustainable resources (electricity from solar panels and energy from halogen light) make this platform the most promising for further development as a sustainable and carbon-neutral process for *n*-butanol production.

## Discussion

In recent years, *n*-butanol has been proposed as a superior biofuel due to its higher energy content, lower volatility, and reduced hydrophilicity^4^. Here we produced *n*-butanol by introducing an artificial *n*-butanol biosynthesis pathway^34^ into an anoxygenic photoautotroph *Rhodopseudomonas palustris* TIE-1^26^. Using metabolic engineering and novel hybrid bioelectrochemical platforms, we show that TIE-1 can produce *n*-butanol using different carbon sources (organic acids, CO_2_), electron sources [H_2_, Fe(II), a poised electrode], and nitrogen sources (NH_4_^+^, N_2_). More interestingly, TIE-1’s ability to produce *n*-butanol under photoelectroautotrophy using light, electricity, and CO_2_ can be a stepping-stone for future sustainable solar fuel production.

After introducing a codon-optimized *n*-butanol biosynthesis pathway in TIE-1 and its mutants (Nif, Gly, and Phb), we determined *n*-butanol production, acetone production, CCE, and electron conversion efficiency (ECE) towards *n*-butanol of these constructs under both photoheterotrophic and photoautotrophic conditions. Mutants lacking the nitrogen-fixing pathway (Nif-3/Nif-5) (known to affect redox balance in the cell by consuming reducing equivalents^37,39,48^) exhibited a more reduced intracellular environment (indicated by higher CO_2_ fixation)^42^ and produced more *n*-butanol compared to WT-3/WT-5. In contrast, deleting acetyl-CoA-consuming pathways (Gly-3/Gly-5 and Phb-3) led to lower *n*-butanol production. These results show that higher reducing equivalent rather than increased acetyl-CoA availability enhances *n*-butanol production by TIE-1. These results also agree with previous works where redox balance or reducing equivalent availability plays a vital role in *n*-butanol production^33,49^. A closely related strain *R. palustris* CGA009 has been shown to produce *n*-butanol when its biosynthesis was the obligate route for maintaining redox balance during photoheterotrophic growth on *n*-butyrate^33^. Similarly, in *E. coli, n*-butanol production increased when its biosynthesis acted as an electron-sink to rescue cells from redox imbalance^49^. In addition to the presence of higher reducing equivalents, we also observed that low cell growth could be another factor that led to higher titer, efficiency, and productivity. These results agree with a previous study where biomass competes for carbon and electrons with the biosynthesis of *n-*butanol^50^.

We did not observe a consistent trend between the 3-gene cassette and 5-gene cassette for *n*-butanol production. In a previous study on PHB production by TIE-1, we observed that although the expression of *phaA* and *phaB* from the TIE-1 genome did not show significant differences, PHB production was different under various growth conditions. We surmised that this might be due to post-transcriptional differences that likely result in different levels of 3-hydroxybutyryl CoA in the cell^27^. Because 3-hydroxybutyryl-CoA is an intermediate of the *n*-butanol synthesis^6^, the amount of 3-hydroxybutyryl-CoA could affect *n-*butanol production. This would likely make it hard to observe any consistent difference between the -3 and -5 strains.

We expected that the presence of NH_4_^+^ would inhibit the expression of nitrogenase, so nitrogen fixation would not occur, and H_2_ would not produce^48,51,52^. However, we observed that WT-3/WT-5 and Gly-3/Gly-5 produced H_2_ (likely via nitrogenase) despite the presence of NH_4_^+^ (Supplementary Fig. 5e). This was in contrast to the Nif-3/Nif-5, which did not produce H_2_ under any condition, confirming that the observed H_2_ production in the WT and Gly strains is due to nitrogenase activity. The production of H_2_ by nitrogenase is well known in CGA009^38,39,42^. This unexpected nitrogenase activity could have been initiated by the lower NH_4_^+^ concentrations toward the end of the experiment, which might lead to the induction of nitrogenase gene expression^51,53^. The Nif mutant does not fix N_2_ or produce H_2_ via the nitrogenase under both non-nitrogen (i.e., with NH_4_^+^) and nitrogen-fixing conditions. This potentially relieves reducing equivalents (NADH) for *n*-butanol production when compared to the WT and the Gly mutants. In addition, the Nif mutant, when incubated under nitrogen-fixing conditions, is a non-growing strain, and this could also account for the higher *n*-butanol production in this mutant under these conditions.

We also observed that by feeding intermediates of *n*-butanol biosynthesis pathway such as 3Hy as a carbon source, TIE-1 produces more *n*-butanol (Fig. 2). However, despite high *n*-butanol production, there was a low CCE and low ECE towards *n*-butanol (Figs. 5, 6), possibly due to higher acetone production (Fig. 4). This high acetone production is likely due to the accumulation of acetoacetyl-CoA, converted from 3Hy through 3-hydroxybutyryl-CoA (Supplementary Fig. 4)^54^. This acetone production, along with 3Hy being an expensive feedstock compared to CO_2_ for bioproduction^55,56^ makes it an unsuitable substrate for economic *n*-butanol production.

In general, we achieved higher *n*-butanol production, CCE, and ECE towards *n*-butanol when acetone production was lower. This is in line with the previous studies where an increase in *n*-butanol production accompanies a decrease in acetone production^57,58^. Although using highly reduced substrates, such as glycerol, can increase the ratio of *n*-butanol to acetone, a significant amount of acetone is always detected while using the *n*-butanol biosynthesis pathway from *C. acetobutylicum*^57,58^. Our study addressed this issue by using slow or non-growing cells that produced *n*-butanol without the production of acetone.

BEC platforms powered by the potentiostat resulted in higher EECE and ECE towards *n*-butanol. This difference is likely due to the different electron uptake control processes of potentiostat Vs. solar panel system. When potentiostat was used, the potential between the cathode and anode was steadily controlled throughout the experiment with respect to the reference electrode. In a solar panel system, the voltage may not be steady, and the current uptake varies with various factors (light intensity, nature of component in the solar system by the manufacture) in addition to the microbial environment^59–61^. As shown in Supplementary Fig. 6a and c, the two electricity sources resulted in huge differences in the total posed current on each system. In addition, the electrical or optical losses associated with the solar panel during photoelectron generation could also affect the effeciency^59,60^. The electrical loss could be due to the limited energy efficiency of the solar panel, which is determined by the diode characteristics and series resistances in the solar panel^59,60^. And optical loss can be in the form of poor light absorbance or light reflection from the solar cell surfaces or material defects^61,62^. We found that the platforms with halogen as the light source have higher EECE towards *n*-butanol (~8-fold) regardless of the electricity source.

To contextualize our results, we compared CCE, EECE, *E*_appl_, and *n*-butanol production with the previous related studies.

### EECE

Using solar panel-generated electricity, TIE-1 achieved an EECE towards *n*-butanol up to 9.54%, which increased by over 13-fold (up to 131.13%) when we used grid-based electricity (Fig. 8f). In a previous study using a hybrid water-splitting system, *R. eutropha* achieved an EECE of 16% towards C_4_ + C_5_ alcohol using grid-based electricity^19^. These data suggest that TIE-1 can also achieve higher EECE using grid-based electricity.

### *E*_appl_ and power requirement

TIE-1 can gain electrons directly from an electrode, which requires lower *E*_appl_ for photoautotrophic growth and *n*-butanol biosynthesis (*E*_app_ = 0.1–0.5 V). In contrast, the hybrid water-splitting system used to synthesize C_3_–C_5_ alcohol or PHB by *R. eutropha* used an *E*_appl_ of 2.0 V. Similarly, *n*-butanol synthesis by *Clostridium* sp. using MES used an *E*_appl_ of 0.8 V^12,19^. Assuming that all the reactors use 1 mA of current, the power would be 5 × 10^-4 ^W for *n*-butanol bioproduction by TIE-1. In contrast, *R. eutropha* would require 2 × 10^−3 ^W for water-splitting, and *Clostridium* sp. would require 8 × 10^−4 ^W. Therefore, TIE-1 uses four times less power than *R. eutropha* and 1.6 times less power than *Clostridium* sp. This implies that even low-efficiency solar panel-based platforms^62^ and low sunlight conditions can be easily used for bioproduction using TIE-1^63,64^.

### n-Butanol production

Under photoelectroautotrophy, TIE-1 produced 0.91 ± 0.07 mg/L of *n*-butanol in 10 days (Fig. 8b). *Clostridium* sp. produced 135 mg/L *n*-butanol in 35 days^12^. Compared to *R. eutropha* and *Clostridium* sp., our platform produced lower *n*-butanol. Under photoautotrophic conditions, TIE-1 produced a maximum of 3.09 ± 0.25 mg/L of *n*-butanol in batch culture (Fig. 2c). Initial studies in cyanobacteria resulted in 2.2 mg/L^14^. Recently, using a modular engineering method, cyanobacteria produced 4.8 g/L of *n*-butanol^17^, which is 2000-fold higher than our initial *n*-butanol production. We anticipate that with intensive future engineering efforts, we can increase the *n*-butanol production efficiency of TIE-1.

### CCE

To the best of our knowledge, no autotrophic *n*-butanol production study has reported CO_2_ consumption^14–17,35,36^. Thus, here we compared TIE-1’s CCE towards *n*-butanol with that reported for heterotrophic production. Although most heterotrophic growth media use yeast extract (an undefined carbon source), for simplicity, our CCE calculations considered the total amount of sugar as the only carbon source for *E. coli* and yeast from the previous studies^9,65^. The early trials in *E. coli* and *S. cerevisiae* reached CCEs of 0.11 and 0.02%^9,65^. As yeast extract also provides carbon, the real CCEs from these studies should be even lower. With intensive metabolic engineering, the CCE towards *n*-butanol reached up to 45.92% in *E. coli* and 11.52% in *S. cerevisiae* (calculated from the reported g/g yield)^34,66^. Our results show that TIE-1 can have the CCE (mol/mol) of up to 4.58 ± 0.21% and 1.95 ± 0.26% under photoautotrophic and photoheterotrophic conditions, respectively (Fig. 5). This is 20 and 200 times higher than that of initial studies in *E. coli* and *S. cerevisiae*. Photoautotrophic bioproduction is superior due to the low cost of CO_2_ compared to heterotrophic substrates^56^. Thus, developing TIE-1 further via metabolic engineering, synthetic biology, and bioprocess engineering will make it an economically viable bioproduction platform.

In summary, TIE-1 can achieve high EECE and CCE towards *n*-butanol with lower power input while producing an amount of *n*-butanol comparable to the initial studies in established bioproduction chassis organisms like *E. coli* and *S. cerevisiae*. This study represents the initial effort of producing carbon-neutral fuels using TIE-1. Although the production is relatively low compared to other model organisms, a number of modifications could be made to improve the *n*-butanol titer. For example, we observed an increased expression of genes in the *n*-butanol biosynthesis pathway from Nif-5 incubated with 3Hy (the strain and condition that resulted in the highest *n*-butanol production) (Supplementary Fig. 8). Therefore, increasing gene expression by driving each gene in the *n*-butanol biosynthesis pathway with its own promoter could increase *n*-butanol production. One of the limitations we face is that only a small portion of electrons go toward *n*-butanol synthesis. Deleting more pathways that consume electrons could make *n*-butanol a more significant electron sink. Also, increasing intracellular iron could lead to higher cytochrome production, which would increase electron uptake^67^. Furthermore, creating a BEC platform with built-in solar conversion to electricity capability could reduce electrical energy loss. Finally, higher electron uptake, which should be beneficial for *n*-butanol synthesis, could be achieved by using nanoparticle-modified electrodes^31,68,69^. Taken together, TIE-1 offers a sustainable route for carbon-neutral *n*-butanol biosynthesis and other value-added products. As CO_2_ concentrations are rising in the atmosphere, such bioproduction strategies need immediate attention and support.

## Methods

### Bacterial strains, media, and growth conditions

All strains used in this study are listed in Supplementary Table 8*E. coli* strains were grown in lysogeny broth (LB; pH 7.0) at 37 °C. For aerobic growth, *Rhodopseudomonas palustris* TIE-1 was grown at 30 °C in YP medium (3 g/L yeast extract, 3 g/L peptone) supplemented with 10 mM MOPS [3-N (morpholino) propanesulphonic acid] (pH 7.0) and 10 mM succinate (YPSMOPS) under the illumination of an infrared LED (880 nm). For growth on a solid medium, YPSMOPS or LB was supplemented with 15 g/L agar. For anaerobic phototrophic growth, TIE-1 was grown in anoxic bicarbonate buffered freshwater (FW) medium^27^. All FW media was prepared under a flow of 34.5 kPa N_2_ + CO_2_ (80%, 20%) and dispensed into sterile anaerobic Balch tubes. The cultures were incubated at 30 °C in an environmental chamber fitted with an infrared LED (880 nm). For photoheterotrophic growth, the FW medium was supplemented with 50 mM MOPS at pH 7.0 and sodium 3-hydroxybutyrate or sodium acetate at pH 7.0, to a final concentration of 50 mM. For photoautotrophic growth on iron, anoxic sterile stocks of FeCl_2_ and nitrilotriacetic acid (NTA) were added to reach final concentrations of 5 mM and 10 mM, respectively. For photoautotrophic growth on H_2_, TIE-1 was grown in FW medium at pH 7.0 and 12 psi of 80% H_2_/20% CO_2_^27^. For all carbon and electron sources, either ammonium chloride (5.61 mM) or dinitrogen gas (8 psi) was supplied as nitrogen source^27^. All sample manipulations were performed inside an anaerobic chamber with a mixed gas environment of 5% H_2_/75% N_2_/20% CO_2_ (Coy Laboratory, Grass Lake, MI). When needed, 400 μg/mL kanamycin was added for TIE-1, and 50 μg/mL kanamycin was added for *E. coli*.

### *R. palustris* TIE-1 deletion mutant construction

We constructed three mutants, two of which were double mutants using the method described in a previous study^30^. Respectively, Glycogen synthase knockout was created by deleting Rpal_0386 (*glgA*), nitrogenase knockout was created by deleting Rpal_5113 (*nifA1*), and Rpal_1624 (*nifA2*), and hydroxybutyrate polymerase knockout was created by deleting and Rpal_2780 (*phaC1*) and Rpal_4722 (*phaC2*) were deleted resulting in hydroxybutyrate polymerase knockout. Briefly, the 1 kb upstream and 1 kb downstream regions of the gene were PCR amplified from the *R. palustris* TIE-1 genome, then the two homology arms of the same gene were cloned into pJQ200KS plasmid. The resulting vector was then electroporated into *E. coli* and then conjugated to *R. palustris* TIE-1, using the mating strain *E. coli* S17-1/λ. After two sequential homologous recombination events, mutants were screened by PCR, as shown in Supplementary Fig. 9. The primers used for mutant construction and verification are listed in Supplementary Tables 9 and 10.

### Plasmid construction

All plasmids used in this study are listed in Supplementary Table 11. There are five genes involved in the *n*-butanol biosynthesis: *phaJ*, *ter*, *adhE2*, *phaA*, and *phaB* (Fig. 1a). Among these five genes, TIE-1 has homologs of the first two (*phaA* and *phaB*). Hence, we designed two different cassettes, namely, a 3-gene cassette (3-gene), which has *phaJ*, *ter*, *adhE*2, and a 5-gene cassette (5-gene), which has the 3-gene plus a copy of the *phaA-phaB* operon from TIE-1. *phaJ*, *ter*, and *adhE2* sequences were obtained from published studies^34^. The *phaJ* gene, isolated from *Aeromonas caviae*, was chosen because it codes for an enzyme that has a higher specificity for its substrate^34,70^. The *ter* gene isolated from *Euflena gracilis* was selected because it is unable to catalyze the reverse oxidation of butyryl-CoA^34^. The *adhE*2 gene isolated from *C. acetobutylicum* is chosen because the enzyme encodes for specifically catalyzes the reduction of the butyryl-CoA^34^. All three foreign genes (*phaJ*, *ter*, and *adhE2*) were codon-optimized by Integrated DNA Technology (IDT) for TIE-1. The cassette was synthesized as G-blocks by IDT, which we then stitched together by overlap extension and restriction cloning. The *phaJ-ter-adhE2* cassette was then inserted into plasmid pRhokS-2, resulting in pAB675. *PhaA* and *phaB* were amplified as an operon from the *R. palustris* TIE-1 genome. The *phaA-phaB* cassette was then cloned into pAB675 to obtain pAB744. Upon obtaining mutants and plasmids, either the 3-gene or the 5-gene was conjugated into WT TIE-1 or the mutants, using mating the strain *E. coli* S17-1/ λ. All conjugations were successful, except for the 5-gene into the Δ*phaC*1Δ*phaC*2. The primers used for cassette construction are listed in Supplementary Table 12. The primers used for cassette sequencing are listed in Supplementary Table 13.

### Substrate measurement

Substrate concentrations at the beginning (T_0_) and the end (T_f_) were measured to calculate carbon and electron conversion efficiency to *n*-butanol. The incubation time of each experiment can be found in Supplementary Table 3.

#### CO_2_ and H_2_ analysis by gas chromatography

CO_2_ and H_2_ were analyzed using a method described in a previous study^27^. Gas samples were analyzed using gas chromatography (Shimadzu BID 2010-plus, equipped with Rt^®^-Silica BOND PLOT Column, 30 m × 0.32 mm; Restek, USA) with helium as a carrier gas. To measure the CO_2_ content of the liquid phase, 1 mL of the cell-free liquid phase was added to 15 mL helium-flushed septum-capped glass vials (Exetainer, Labco, Houston) containing 1 mL 85% phosphoric acid. Then 40 μL of the resulting gas from the Balch tube was injected into the Shimazu GC-BID, using a Hamilton^TM^ gas-tight syringe. To measure the CO_2_ and H_2_ contents of the gas phase, either 40 μL of the gas phase was directly injected into the Shimadzu GC-BID, or 5 mL of the gas phase was injected into a 15 mL helium-flushed septum-capped glass vial (Exetainer, Labco, Houston), using a Hamilton^TM^ gas-tight syringe. Then 50 μL of the diluted gas sample was injected into the Shimazu GC-BID, using a Hamilton^TM^ gas-tight syringe. A standard curve was generated by the injection of 10 μL, 25 μL, and 50 μL of H_2_ + CO_2_ (80%, 20%). The total moles of CO_2_ in the reactors were calculated using the ideal gas law (PV = nRT)^71^.

#### Organic acid analysis by ion chromatography

For measuring organic acid concentration, after 1:50 dilution, the acetate and 3-hydroxybutyrate concentrations at the starting and endpoint of culture for each sample were quantified using an Ion Chromatography Metrohm 881 Compact Pro with a Metrosep organic acid column (250 mm length). Eluent (0.5 mM H_2_SO_4_ with 15% acetone) was used at a flow rate of 0.4 mL min^−1^ with suppression (10 mM LiCl regenerant)^27^.

#### Ferrous iron [Fe(II)] analysis by ferrozine assay

The Fe(II) concentration measurement was done using 10 μL of culture mixed with 90 μL 1 M HCl in a 96-well plate inside the anaerobic chamber (filled with 5% H_2_/75% N_2_/20% CO_2_, Coy Laboratory, Grass Lake, MI). After the plate was removed from the anaerobic chamber, 100 μL of ferrozine (0.1% (w/v) ferrozine in 50% ammonium acetate) was added to the sample. Then the 96-well plate was covered with foil and incubated at room temperature for 10 min. before the absorbance was measured at 562 nm. The absorbance was then converted to Fe(II) concentration based on a standard curve generated by measuring the absorbance from 0 mM, 1 mM, 2.5 mM and, 5 mM Fe(II).

### In vivo production of *n*-butanol

The plasmids with the *n*-butanol pathway were unstable when adapting the strain to the nitrogen-fixing or photoautotrophic conditions. To avoid this problem, a twice-washed heavy inoculum from YPSMOPS was used under all conditions. All strains were inoculated in 50 mL of YPSMOPS with kanamycin with a 1:50 dilution from a pre-grown culture. When the OD_660_ reached 0.6–0.8, the culture was inoculated into 300 mL of YPSMOPS with kanamycin. When the OD_660_ reached 0.8~1, 10 mL of culture was saved for a PCR check (Supplementary Fig. 10). The rest of the culture was washed twice with ammonium-free FW medium and resuspended using anoxic ammonium-free FW medium inside the anaerobic chamber. Finally, the culture was inoculated into the medium containing different carbon sources and electron donors (acetate, 3-hydroxybutyrate, H_2_, Fe(II), or electrode) in either a sealed Balch tube (initial OD_660_ ~1) or a BEC (initial OD_660_ ~0.7). The tubes and the reactors were sealed throughout the process, and samples were taken after the cultures reached the stationary phase (incubation time listed in Supplementary Table 3), using sterile syringes.

### Extraction and quantification of *n*-butanol and acetone

After the culture entered the late stationary phase, 1 mL of culture was removed from the culture tube using a syringe and centrifuged at 21,100 × *g* for 3 min. The supernatant was then filtered using a syringe filter, and the filtrate or the standard was extracted with an equal volume of toluene (containing 8.1 mg/L iso-butanol as an internal standard) and mixed using a Digital Vortex Mixer (Fisher) for 5 min. followed by centrifugation at 21,100 × *g* for 5 min. After centrifugation, 250 μL of the organic layer was added to an autosampler vial with an insert. The organic layer was then quantified with GC-MS (Shimazu GCMS-QP2010 Ultra), using the Rxi^®^-1ms column. The oven was held at 40 °C for 3 min, ramped to 165 °C at 20 °C/min, then held at 165 °C for 1 min. Samples were quantified relative to a standard curve for 0, 0.2025, 0.405, 0.81, 2.025, 4.05, and 8.1 mg/L of *n*-butanol and 0, 0.784, 3.92, 7.84, 39.2, 78.4, and 392 mg/L of acetone. An autosampler was used to reduce the variance of injection volumes.

### Bioelectrochemical platforms and growth conditions

A three-electrode sealed-type bioelectrochemical cell (BEC, C001 Seal Electrolytic Cell, Xi’an Yima Opto-electrical Technology Com., Ltd, China)^30,68^ containing 80 mL of FW medium was used for testing *n*-butanol production. The three electrodes were configured as a working electrode (a graphite rod, 3.2 cm^2^), a reference electrode (Ag/AgCl in 3.5 M KCl), and a counter electrode (Pt foil, 5 cm^2^). FW medium (76 mL) was dispensed into sterile, sealed, three-electrode BECs, which were bubbled for 60 min. with N_2_ + CO_2_ (80%/20%) to remove oxygen and pressurized to ~7 psi. Four BECs were operated simultaneously (*n* = 3 biological replicates) with one no-cell control. All photoelectroautotrophic experiments were performed at 26 °C under continuous infrared light (880 nm) or halogen light. The electrical potential of 0.5 V (*E*_appl _= 0.5 V) was constantly applied (240 h) to the working electrode with respect to the reference electrode (Ag/AgCl in 3.5 M KCl) and counter electrode using a grid powered potentiostat (Interface 1000E, Gamry Multichannel potentiostat, USA) or The solar panel (Uxcell 0.5 V 100 mA Poly Mini Solar Cell Panel Module) with the output voltage 0.5 V (*E*_appl _= 0.5 V) was directly connected to the bioreactors for 240 h and the resulting current uptake/electron uptake to the bioreactor was measured with the resistor using ohm’s law of electrical current. Electron uptake was collected every 1 min using the Gamry Echem Analyst™ (Gamry Instruments, Warmister, PA) software package. At the end of the bioelectrochemical experiment, the samples were immediately collected from the BEC reactors. *n*-butanol, acetone, and substrates were measured as described above.

### Calculations of CCE, electron conversion efficiency, electrical energy conversion efficiency, and electron consumption of *n-*butanol, acetone, CO_2_, H_2_, and biomass biosynthesis

CCE, electron conversion efficiency, and EECE were calculated by dividing the total carbon/electrons/electrical-energy consumption by the final carbon/electrons/energy content in *n*-butanol, respectively.

To determine carbon consumption, acetate, 3-hydroxybutyrate, or CO_2_ consumption was calculated by subtracting the amount in the sample at the end of the experiment from the amount at the beginning of the experiment. Then all the carbon substrate consumptions were converted to moles of carbon, using Eq. (1). The amount of carbon converted to *n*-butanol was calculated based on the *n*-butanol production, using Eq. (2). The CCE was calculated using Eqs. (1), (2), and (3) below:

1 $${{{{{\rm{C}}}}}}\,{{{{{\rm{mol}}}}}}\,{{{{{\rm{substrate}}}}}}={{{{{\rm{consumed}}}}}}\,{{{{{\rm{substrate}}}}}}\left(\frac{{{{{\rm{mol}}}}}}{L}\right)* {{{{\rm{mol}}}}}\,{{{{{\rm{of}}}}}}\,{{{{{\rm{C}}}}}}\,{{{{{\rm{in}}}}}}\,1\,{{{{{\rm{mol}}}}}}\,{{{{{\rm{substrate}}}}}}$$

2 $${{{{{\rm{C}}}}}}\,{{{{{\rm{mol}}}}}}\,n{\mbox{-}}{{{{{\rm{butanol}}}}}}=\frac{n{\mbox{-}}{{{{{\rm{butanol}}}}}}\,(g/L)* {{{{{\rm{mol}}}}}}\,{{{{{\rm{of}}}}}}\,{{{{{\rm{C}}}}}}\,{{{{{\rm{in}}}}}}\,1\,{{{{{\rm{mol}}}}}}\,n{\mbox{-}}{{{{{\rm{butanol}}}}}}}{{{{{{\rm{molecular}}}}}}\,{{{{{\rm{weight}}}}}}\,{{{{{\rm{of}}}}}}\,n{\mbox{-}}{{{{{\rm{butanol}}}}}}}$$

3 $${{{{{\rm{Carbon}}}}}}\,{{{{{\rm{conversion}}}}}}\,{{{{{\rm{efficiency}}}}}}=\frac{{{{{{\rm{C}}}}}}\,{{{{{\rm{mol}}}}}}\,n{\mbox{-}}{{{{{\rm{butanol}}}}}}}{{{{{{\rm{C}}}}}}\,{{{{{\rm{mol}}}}}}\,{{{{{\rm{sunstrate}}}}}}}* 100 \%$$

The theoretical total number of electrons available from each consumed electron donor was calculated as described below (Eq. (4)). The total available electrons from the complete oxidation of each organic acid were calculated with the assumption that the final oxidation product was CO_2_. The inorganic electron donors such as Fe(II) and H_2_ release 1 mole e^−^ and 2 moles e^−^ per mole, respectively. Electrons supplied for the photoelectroautotrophy condition were calculated directly from BEC-based experiments wherein the total current uptake was integrated over the operational time. The total electron uptake was used to calculate the electron conversion efficiency to *n*-butanol because the electrode is the direct electron donor under this growth condition. The number of electrons required for *n*-butanol production was calculated from the oxidation state of the carbon in each carbon source and *n*-butanol. Supplementary Table 14 lists the specific oxidation state, and the number of electrons required per mole of *n*-butanol is listed for all studied sources and *n*-butanol.

To calculate the total available electrons from each substrate, the amount of consumed substrate (in moles) was multiplied by the theoretical total available electrons per mole of the substrate when fully oxidized to CO_2_ (Eq. (4)). For photoelectroautotrophy, the total available electron was calculated based on data collected from a data acquisition system (DAQ, Picolog Datalogger). To obtain the electrons required for *n*-butanol production, the *n*-butanol production (in moles) was multiplied by the theoretical number of electrons required per mole (Eq. (5)). The conversion efficiency was calculated by dividing the moles of electrons required for *n*-butanol production by the theoretical total available electrons (Eq. (6)).

4 $${e}^{-}{{{{\rm{mol}}}}}\,{{{{{\rm{substrate}}}}}}={{{{{\rm{consumed}}}}}}\,{{{{{\rm{substrate}}}}}}\,({{{{\rm{mol}}}}})* {{{{{\rm{total}}}}}}\,{{{{{\rm{available}}}}}}\,{{{{{\rm{electrons}}}}}}\,{{{{{\rm{in}}}}}}\,{{{{{\rm{the}}}}}}\,{{{{{\rm{substrate}}}}}}$$

5 $${e}^{-}{{{{{\rm{mol}}}}}}\,n{\mbox{-}}{{{{{\rm{butanol}}}}}}=n{\mbox{-}}{{{{{\rm{butanol}}}}}}\,({{{{\rm{mol}}}}})*{{{{{\rm{electrons}}}}}}\,{{{{{\rm{required}}}}}}\,{{{{{\rm{to}}}}}}\,{{{{{\rm{synthesize}}}}}}\,1\,{{{{{\rm{mol}}}}}}\,n{\mbox{-}}{{{{{\rm{butanol}}}}}}$$

6 $${{{{{\rm{Electron}}}}}}\,{{{{{\rm{conversion}}}}}}\,{{{{{\rm{efficiency}}}}}}=({e}^{-}{{{{\rm{mol}}}}}\,n{\mbox{-}}{{{{{\rm{butanol}}}}}})/({e}^{-}{{{{\rm{mol}}}}}\,{{{{{\rm{substrate}}}}}})\,*\, 100 \%$$

Calculation of the EECE to *n*-butanol was adapted from a previous study^19^. The EECE was calculated by Eq. (7). The charge supplied to the bioelectrochemical platforms was calculated from data collected by DAQ.

7 $${{{{{\rm{EECE}}}}}}=\frac{{\varDelta }_{r}{G}^{0}{{{{{\rm{gain}}}}}}\,{{{{{\rm{from}}}}}}\,{{{{{\rm{C}}}}}}{{{{{\rm{O}}}}}}_{2}{{{{{\rm{to}}}}}}\,n{\mbox{-}}{{{{{\rm{butanol}}}}}}}{{{{{{\rm{charge}}}}}}\,{{{{{\rm{passed}}}}}}\,{{{{{\rm{through}}}}}}\,(C)\,*\, {{{{{\rm{applied}}}}}}\,{{{{{\rm{voltage}}}}}}\,(V)}\,*\, 100 \%$$

The Gibbs free energy gains (${\Delta }_{{{{{{\rm{r}}}}}}}{{{{{{\rm{G}}}}}}}^{0}$) for *n*-butanol was calculated similarly with a previous study^19^ by reaction 8 and Eq. (9)^72^.

8 $${{{{{\rm{C}}}}}}_{4}{{{{{\rm{H}}}}}}_{10}{{{{{\rm{O}}}}}}\,({{{{\rm{l}}}}})+6\,{{{{{\rm{O}}}}}}_{2}\to 4\,{{{{{\rm{C}}}}}}{{{{{\rm{O}}}}}}_{2}({{{{\rm{g}}}}})+5\,{{{{{\rm{H}}}}}}_{2}{{{{{\rm{O}}}}}}\,({{{{\rm{l}}}}})$$

9 $${\varDelta }_{r}{G}_{({C}_{4}{{{{{\rm{H}}}}}}_{10}{{{{\rm{O}}}}})}^{0}={\varDelta }_{f}{H}_{({{{{{\rm{C}}}}}}_{4}{{{{{\rm{H}}}}}}_{10}{{{{\rm{O}}}}})}^{0}-5\ast {\varDelta }_{f}{H}_{({{{{{\rm{H}}}}}}_{2}{{{{\rm{O}}}}})}^{0}-\,4\ast {\varDelta }_{f}{H}_{({{{{\rm{C}}}}}{{{{{\rm{O}}}}}}_{2})}^{0}-6\ast {\varDelta }_{f}{H}_{({{{{{\rm{O}}}}}}_{2})}^{0}$$

$${\varDelta }_{f}{H}_{({{{{{\rm{C}}}}}}_{4}{{{{{\rm{H}}}}}}_{10}{{{{\rm{O}}}}})}^{0}=-77.4\,{{{{{\rm{kJ}}}}}}/{{{{{\rm{mol}}}}}},\varDelta {{{{{{\rm{G}}}}}}}_{({{{{\rm{C}}}}}{{{{{\rm{O}}}}}}_{2})}^{0}=-394.39\,{{{{{\rm{kJ}}}}}}/{{{{\rm{mol}}}}},\varDelta {{{{{{\rm{G}}}}}}}_{({{{{{\rm{H}}}}}}_{2}{{{{\rm{O}}}}})}^{0}=-273.14\,{{{{{\rm{kJ}}}}}}/{{{{{\rm{mol}}}}}},\varDelta {{{{{{\rm{G}}}}}}}_{({{{{{\rm{O}}}}}}_{2})}^{0}=0\,{{{{{\rm{kJ}}}}}}/{{{{{\rm{mol}}}}}}$$

Electron consumption of *n*-butanol, acetone, CO_2_, H_2_, and biomass biosynthesis, were calculated by the mole of production multiplied by the electrons required for each mole of product. The molar production of *n*-butanol and acetone was determined by the titer divided by molecular weight. The molar production of CO_2_, H_2_, was measured by GC-BID. The molar production of biomass was calculated by the OD_660_ change between T_0_ and T_f_ by Eq. (10).

10 $${{Molar}}\,{{production}}\,{{of}}\,{{biomass}}=\frac{({O}{{D}}_{Tf}-{O}{{D}}_{T0})* (\frac{{cell}\,{number}}{ml})* {{cell}}\,{{weight}}}{{Molecular}\,{weight}\,{of}\,{biomass}}$$

Cell number/ml: 8 × 10^8^ cell/ml/OD, cell weight: 10^−12 ^g/cell^73^, Molecular weight of biomass: 22.426 g/mol^42^.

### Determination of glycogen content

TIE-1 cells were grown in freshwater medium with NH_4_Cl or under nitrogen-fixing condition (with N_2_) supplemented with 10 mM 3-hydroxybutyrate to and OD_660_ of 1.8 mL of bacterial culture was pelleted and washed three times with ultrapure water and resuspended in 30% (w/v) KOH with for glycogen extraction. Samples were then incubated at 95 °C for 2 h. Glycogen was precipitated by the addition of ice-cold ethanol to a final concentration of 75%. Samples were put on ice for 2 h followed by 10 min. centrifugation 10,000 × *g* at 4 °C. The precipitated glycogen was then washed twice with pure ethanol and dried for 20 min. at 60 °C. Glycogen samples were resuspended in 250 µL of 100 mM sodium acetate (pH 4.5) and digested with 2 mg/ml amyloglucosidase (Sigma Aldrich A7420) for 2 h at 60 °C. Samples were added with infinity glucose hexokinase liquid reagent (Thermo scientific TR1542) at a ratio of 1:150 according to the manufacturer’s recommendation and absorbance reading was done at 340 nm^74^.

### RNA extraction, cDNA synthesis, and RT-qPCR

To extract RNA for cDNA synthesis and eventually perform RT-qPCR for analyzing the expression level of the individual genes, culture samples (2.5 ml to 15 ml depending on OD_660_) were taken at the late exponential (*T*_m_) or stationary phase (*T*_f_). Samples were immediately stabilized with an equal volume of RNAlater (Qiagen, USA). After incubation at room temperature for 10 min, samples were centrifuged at 21,100 × *g* for 3 min. After the supernatant was removed, the pellet was stored at −80 °C before RNA extraction using the Qiagen RNeasy Mini kit (Qiagen, USA), following the manufacturer’s protocol. DNA was removed using a Turbo DNA-free Treatment and Removal Kit (Ambion, USA). DNA contamination was ruled out by PCR using the primers listed in Supplementary Tables 12 and 13.

Purified RNA samples were then used for cDNA synthesis by an iScript^TM^ cDNA Synthesis Kit (Biorad, USA). The same mass of RNA was added to each cDNA synthesis reaction. The synthesized cDNA was used for RT-qPCR. RT-qPCR was performed using the Biorad CFX connect Real-Time System Model # Optics Module A with the following thermal cycling conditions: 95 °C for 3 min, then 30 three-step cycles of 95 °C for 3 s, 60 °C for 3 min, and 65 °C for 5 s, according to the manufacturer’s manual. The reaction buffer was iTaq SYBR Green Supermix with ROX (Bio-Rad). The primers used for RT-qPCR (listed in Supplementary Table 15) were designed using primer3 software (http://bioinfo.ut.ee/primer3/). The primer efficiencies were determined by performing RT-qPCR using different DNA template concentrations. The genes *clpX* and *recA*, which have been previously validated as internal standards, were used^29,30^. The gene code for kanamycin resistance was also used as an internal standard for the plasmid. After RT-qPCR, the data were analyzed using the ΔΔC_T_ method.

### NADH/NAD^+^ measurement

Wild-type TIE-1 and Nif mutants were grown with either 3-hydroxybutyrate or H_2_ and using NH_4_Cl as a nitrogen source. Briefly, 1.8 mL cell cultures from the stationary phase (same phase at which samples were taken for measuring *n*-butanol) was spined at 21,000 × *g* for 1 min. inside an anaerobic chamber. Then the pellet was resuspended in either 300 μL 0.2 M sodium hydroxide (for NADH extraction) or 300 μL 0.2 M hydrochloric acid (for NAD^+^ extraction). The resuspension was then incubated at 50 °C for 10 min. and cooled to below 20 °C on an ice block. While vortexing on medium speed, Equal volume 0.1 M acid or base was added to neutralize the sample. After spinning at 21,000 × *g* for 5 min, the supernatant was stored in freezer for the following assays. The enzyme cycling assays were then performed on a BioTek Synergy^TM^ HTX 96-well plate reader measuring absorbance at 570. The amount of NADH/NAD^+^ was quantified relative to a standard curve ranging from 0 to 5 μM.

### Transmission electron microscopy (TEM)

Wild-type TIE-1 and Phb mutants grown with 3-hydroxybutyrate with either N_2_ or NH_4_Cl as a nitrogen source was used as representative samples for TEM. Briefly, 5 mL planktonic cell suspensions were centrifuged at 6000×g for 5 min. followed by primary fixation by resuspending the cell pellets in 2% formaldehyde and 2.5% glutaraldehyde in 0.05 M sodium cacodylate buffer (pH 7.2) for ~45 min. at room temperature. Cell pellets were agar encapsulated followed by primary fixation for ~20 min. Polymerized agar was cut into small cubes and were subjected to secondary fixation for ~5 h followed by acetone dehydration and resin infiltration. Ultrathin sections (~70 nm) were cut on a Reichert Ultracut UCT ultramicrotome (Leica, Buffalo Grove, IL, USA), mounted on copper grids (FCFT300-CU-50, Electron Microscopy Sciences, Hatfield, PA, USA), and counterstained with lead citrate for 8 min^75^. The sample was imaged with a LEO 912 AB Energy Filter Transmission Electron Microscope (Zeiss, Oberkochen, Germany). Images were acquired with iTEM software (ver. 5.2) (Olympus Soft Imaging Solutions GmbH, Germany) with a TRS 2048 × 2048k slow-scan charge-coupled device (CCD) camera (TRÖNDLE Restlichtverstärkersysteme, Germany). Each TEM image was acquired at ×10,000 magnification and 1.37 nm pixel resolution.

### Viability analysis of TIE-1 under photoelectroautotrophy

WT TIE-1 was inoculated into the bioelectrochemical reactors described above, with a starting OD of ~0.3. After 72 h of incubation, the viability of the biofilm attached to the electrode was characterized by imaging the electrode after staining with the LIVE/DEAD^®^ (L7012, Life Technologies) kit. The attached cells were quantified using NIS-Elements AR Analysis 5.11.01 64-bit software. For imaging of the electrode, prior to cutting a piece of the spent electrode, the electrode from the reactor was washed three times with 1× phosphate-buffered saline (PBS) to remove unattached cells. A piece of the spent electrode was then submerged in 1× PBS in a sterile microfuge tube. Prior to imaging, the electrode piece was immersed in LIVE/DEAD^®^ stain (10 μM SYTO9 and 60 μM propidium iodide) kit and incubated for 30 min. in the dark. The electrode sample was then placed in a glass-bottom Petri dish (MatTek Corporation, Ashland, MA) containing enough PBS to submerge the sample. Further, it was imaged on a confocal microscope (Nikon A1 inverted confocal microscope), using 555 and 488 nm lasers and a ×100 objective lens (Washington University in St. Louis Biology Department Imaging Facility). Electrode attached cells were quantified by Elements Analysis software using the protocol described below: Briefly, for each reactor, three images were processed. Z-stacks of each image were split into two channels (one for live cells, one for dead cells), the MaxIP was acquired for the combined z-stacks. After GaussLaplace, local contrast and smoothing, and thresholding, and Object Count was performed for each channel based on a defined radius (0.8–5 μm). Then the percentage of live (or dead) cells was calculated by

$${{{{{\rm{Live}}}}}}\,({{{{{\rm{or}}}}}}\,{{{{{\rm{Dead}}}}}})\,{{{{{\rm{cell}}}}}}\,{{{{{\rm{percentage}}}}}}=\frac{{{{{{\rm{number}}}}}}\,{{{{{\rm{of}}}}}}\,{{{{{\rm{Live}}}}}}\,({{{{{\rm{or}}}}}}\,{{{{{\rm{Dead}}}}}})\,{{{{{\rm{cells}}}}}}}{{{{{{\rm{number}}}}}}\,{{{{{\rm{of}}}}}}\,{{{{{\rm{total}}}}}}\,{{{{{\rm{cells}}}}}}}\,*\, 100 \%$$

### Toxicity study

WT TIE-1 with an empty vector (pRhokS-2) was used to test the tolerance of TIE-1 for acetone and *n*-butanol. To test the tolerance, 0, 0.25, 0.5, 1, or 2% *n*-butanol (v/v), or 0, 0.1, 0.25, 0.5, 1, or 2% acetone (v/v), was added to FW media with acetate (10 mM). Growth was monitored by recording OD_660_ over time.

### Statistics

All statistical analyses (two tails Student’s *t*-test) were performed with Python. *p*-value < 0.05 was considered to be significant. *p*-values are presented in supplementary data 2, and estimated effect size (Cohen’s *d*) are presented in supplementary data 3. For most of the experiments data are from *n* = 3 of biologically independent samples, from each biologically independent samples *n* = 3 technical replications were performed, For photoelectroautotrophy measurements, data are from *n* = 2 of biologically independent samples, from each biologically independent samples *n* = 3 technical replications were performed. For RT-qPCR for photoelectroautotrophy which data are from *n* = 2 of biologically independent samples, from each biologically independent sample *n* = 2 technical replications were performed.

### Reporting summary

Further information on research design is available in the Nature Research Reporting Summary linked to this article.

## Supplementary information

Supplementary Information

Description of Additional Supplementary Files

Supplementary Data 1 - Source data

Supplementary Data 2 - p-values table

Supplementary Data 3 - effective size

Reporting Summary

## Data Availability

All the source data for the main figures are included in the supplementary data. All data in this study are available from the corresponding authors upon request. We have deposited all plasmids to Addgene. Also, all plasmids will be made available upon request.
