# Supplementary material for: n-Butanol production by Rhodopseudomonas palustris TIE-1
Source: Commun Biol. 2021 Nov 3;4:1257. doi: 10.1038/s42003-021-02781-z (PMC8566592; doi:10.1038/s42003-021-02781-z)
Supplement: Supplementary file 1 — Supplementary Information [file 42003_2021_2781_MOESM1_ESM.pdf]

Supplementary Information

n-Butanol production by *Rhodopseudomonas palustris* TIE-1

Bai, et al.

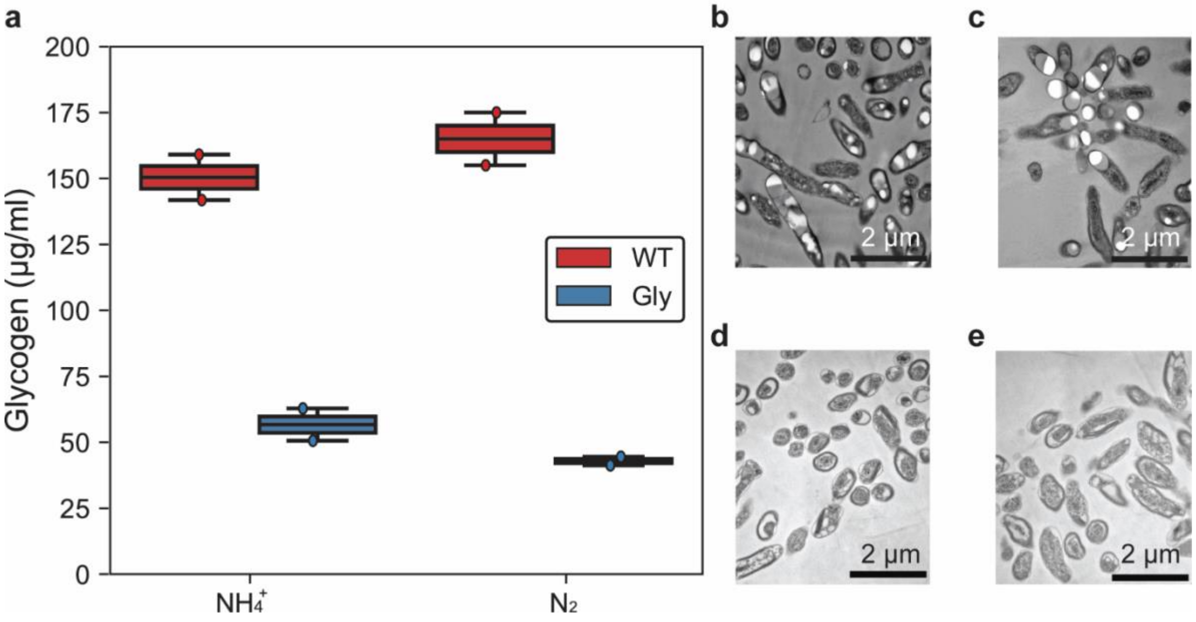

Supplementary Figure 1. Polyhydroxybutyrate and glycogen accumulation by mutants and the wild-type strain. (**a**) Glycogen content of WT and Gly mutant grown with NH_4^+ or N_2 as a nitrogen source. (**b-e**) TEM micrographs of polyhydroxybutyrate granulate (bright spots in the figures) in WT and Phb mutant. (**b**) WT with N_2, (**c**) WT with NH_4^+, (**d**) Phb with N_2, (**e**) Phb mutant with NH_4^+. 3-Hydroxybutyrate was used as a carbon source for all the growth conditions. Data are from *n*=3 of independent experiments. WT: wild type; Gly: glycogen synthase knockout; Phb: 3-hydroxybutyrate polymerase knockout

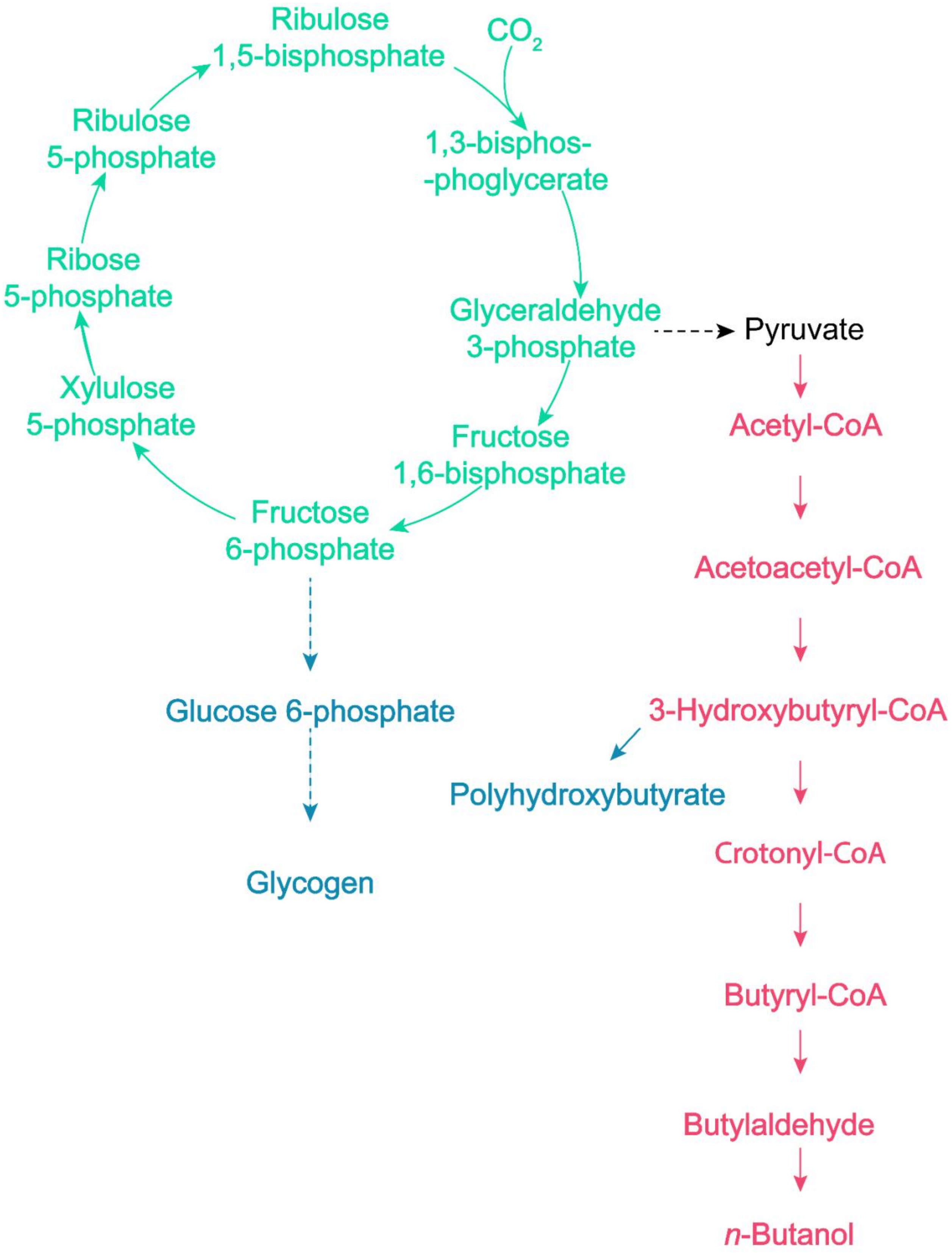
Ribulose
1,5-bisphosphate
CO_2
1,3-bisphos-
-phoglycerate
Glyceraldehyde
3-phosphate
Pyruvate
Fructose
1,6-bisphosphate
Fructose
6-phosphate
Xylulose
5-phosphate
Ribose
5-phosphate
Ribulose
5-phosphate
Glucose 6-phosphate
Glycogen
Acetyl-CoA
Acetoacetyl-CoA
3-Hydroxybutyryl-CoA
Polyhydroxybutyrate
Crotonyl-CoA
Butyryl-CoA
Butylaldehyde
n-Butanol

Supplementary Figure 2. Relationship between polyhydroxybutyrate, glycogen, and *n*-butanol biosynthesis pathways and Calvin-Benson-Bassham cycle. Pink: *n*-butanol biosynthesis pathway, Green: Calvin-Benson-Bassham cycle, Blue: glycogen and polyhydroxybutyrate biosynthesis.

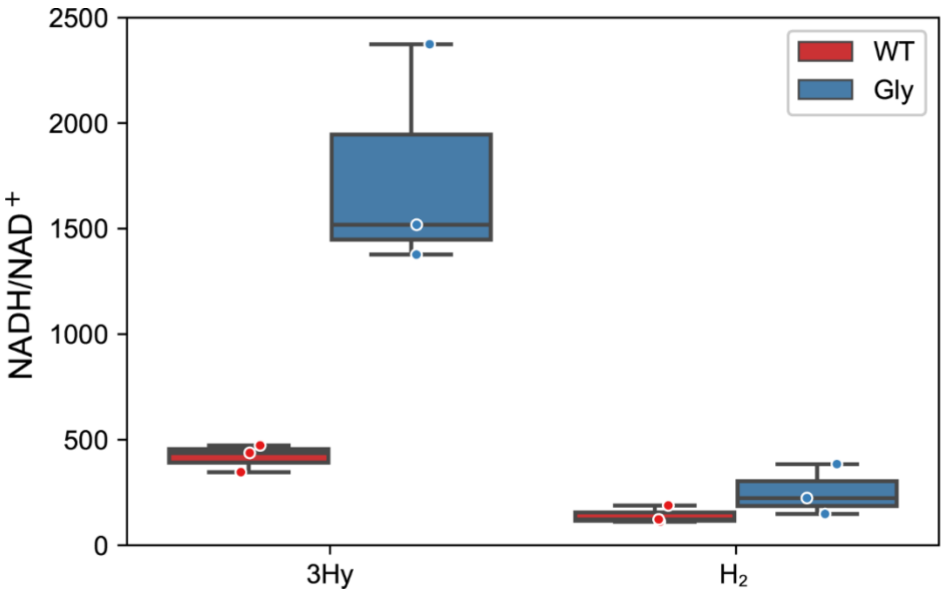

Supplementary Figure 3. The NADH/ NAD^+ ratio of nitrogenase knockout and wild-type strain. Data are from *n*=3 of independent experiments. WT: wild type, Nif: nitrogenase knockout, 3Hy: using 3-hydroxybutyrate as carbon and electron source, H_2: using hydrogen as an electron source, CO_2 as carbon source. All growth used NH_4^+ as the nitrogen source.

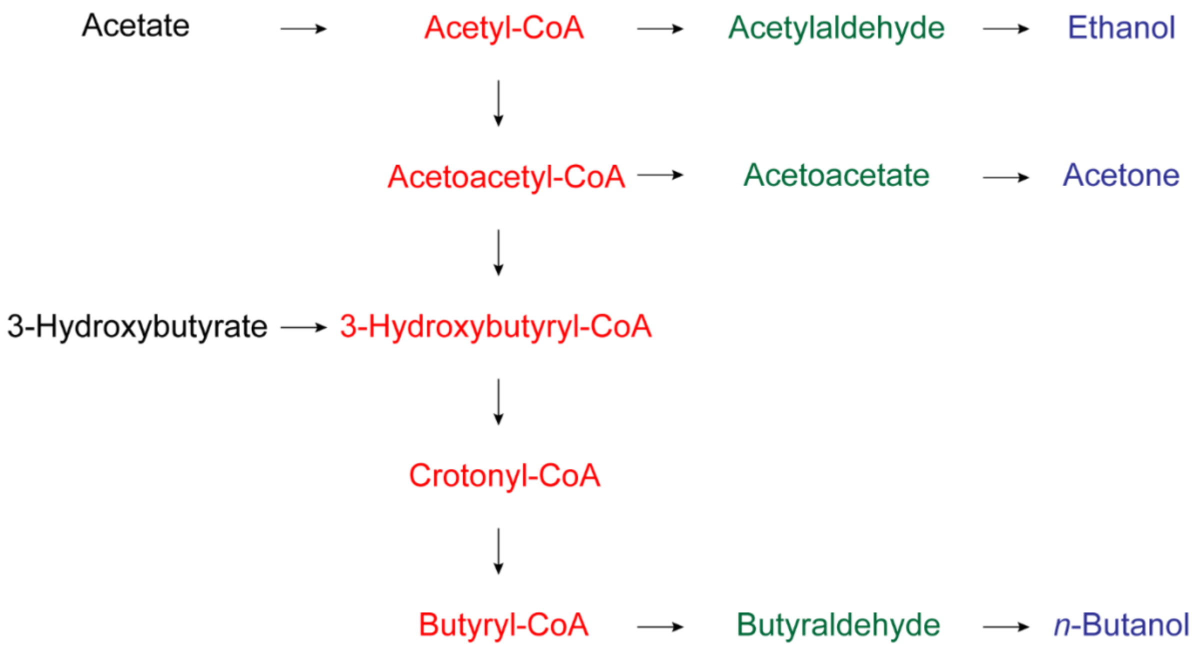

Supplementary Figure 4. Substrates and by-products of *n*-butanol biosynthesis. Black: substrates; Red: *n*-butanol synthesis intermediates; Green: in-cell by-products; Blue: secreted by-products.

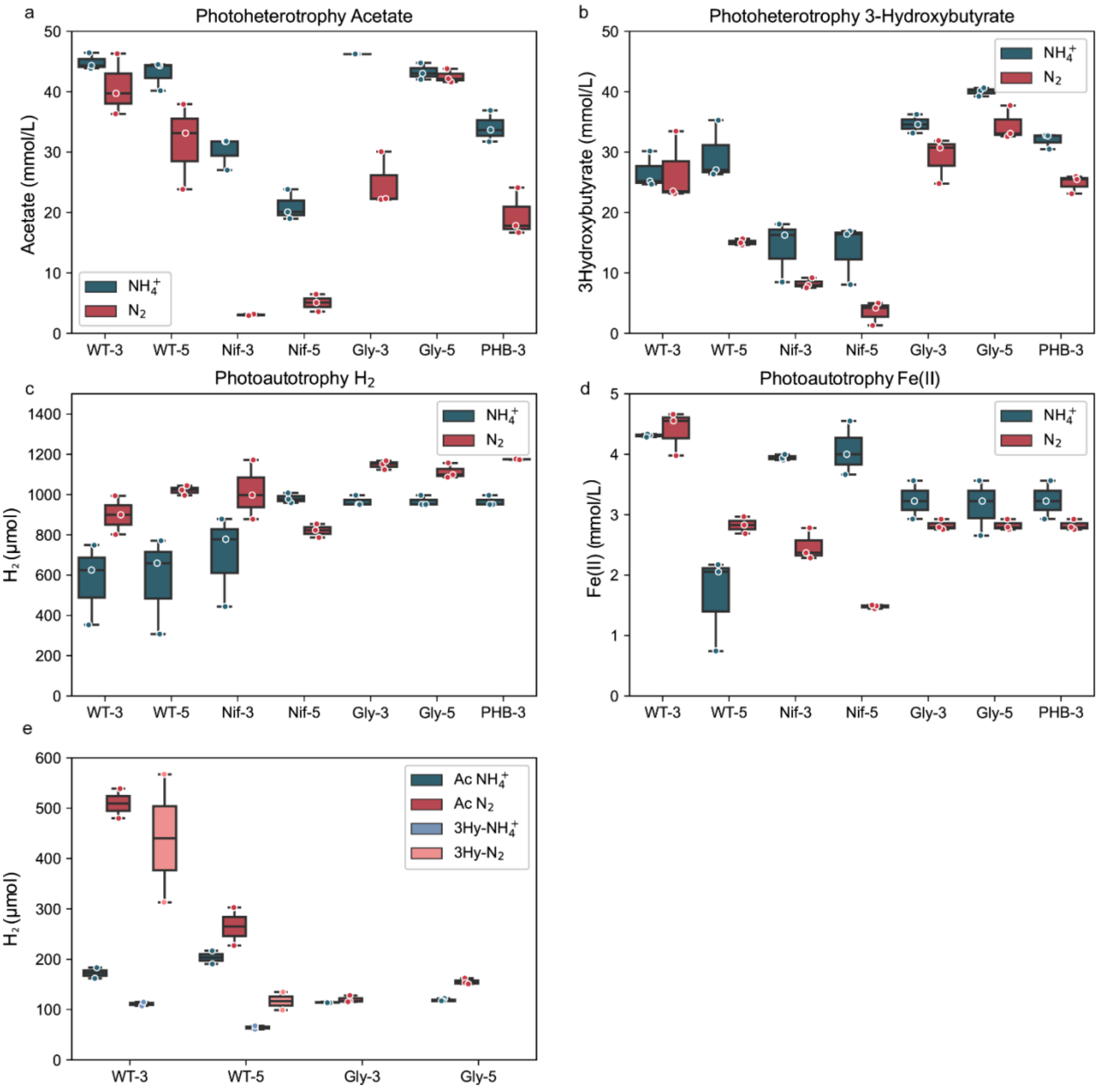

Supplementary Figure 5. Substrate consumption and hydrogen production under photoheterotrophic conditions. (**a-d**) Electron donor consumption when TIE-1 was cultured with ammonium (NH_4^+, red) or dinitrogen gas (N_2, blue); (**a**) acetate (photoheterotrophy); (**b**) 3-hydroxybutyrate (photoheterotrophy); (**c**) hydrogen (H_2) (photoautotrophy); (**d**) ferrous iron (Fe(II)) (photoautotrophy); (**e**). H_2 production of WT-3/WT-5 and Gly-3/Gly-5 mutant under photoheterotrophic conditions. Data are from *n*=3 of independent experiments. Carbon dioxide was present in all conditions. CO_2: carbon dioxide; WT-3: wild type with 3-gene cassette; WT-5: wild type with 5-gene

cassette; Nif-3: nitrogenase knockout t with 3-gene cassette; Nif-5: nitrogenase knockout with 5-gene cassette; Gly-3: glycogen synthase knockout with 3-gene cassette; Gly-5: glycogen synthase knockout with 5-gene cassette; Phb-3: hydroxybutyrate polymerase knockout with 3-gene cassette, n.d. (non-detectable).

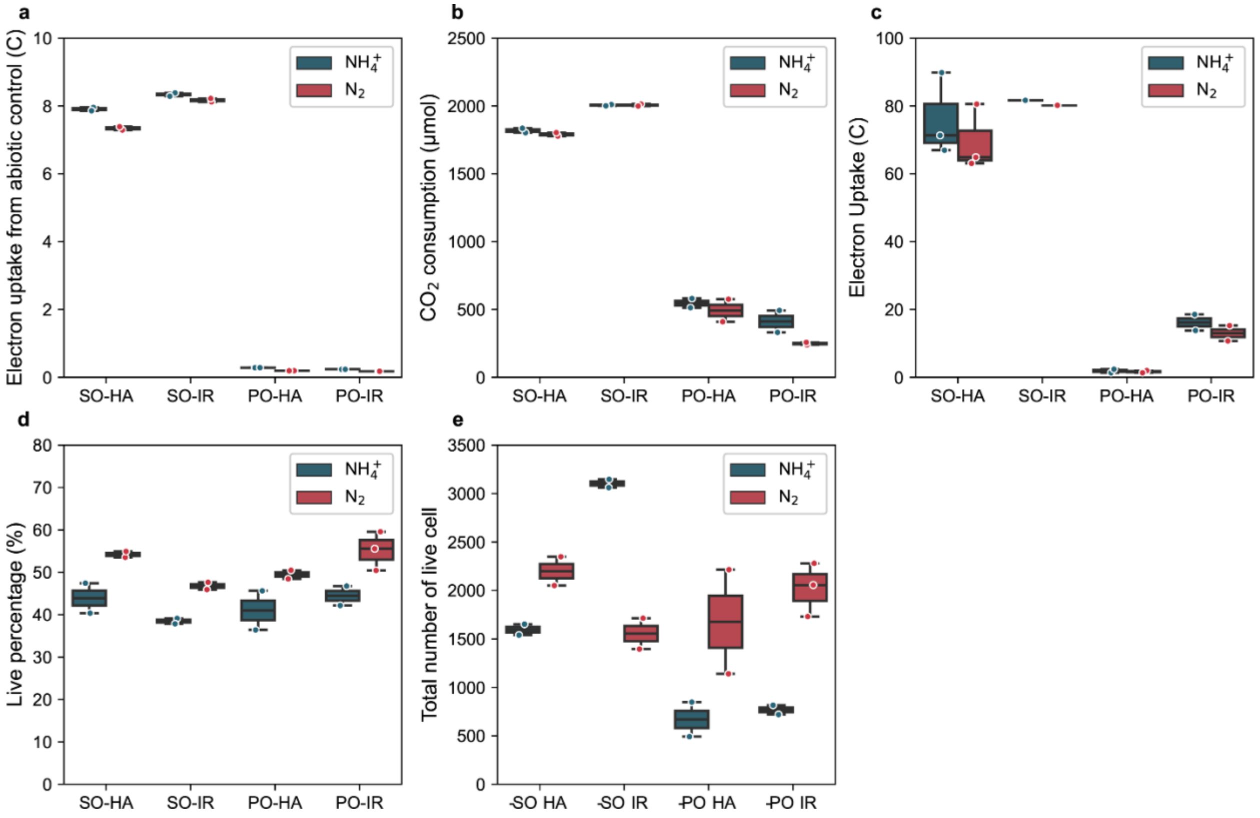

Supplementary Figure 6. Electron uptake of abiotic control, carbon consumption, electron uptake, cell viability in each reactor setup. a. Electron uptake in abiotic control **b**. Carbon dioxide consumption **c**. Current uptake **d**. The percentage of live cells **e**. The total number of live cells in the presence of ammonium (NH_4^+, red) or dinitrogen gas (N_2, blue) with various light and electricity sources. Data are from *n*=2 of independent experiments. SO: using electricity generated by a solar panel; HA: using halogen light as the light source. PO: using electricity from potentiostat as the electricity source; IR: using infrared light as the light source. NH_4^+: ammonium; N_2: dinitrogen gas. Exp: WT TIE-1 with illumination and closed-circuit passing current; Dark: dark control group using WT TIE-1 without illumination; OC: open circuit control, WT TIE-1 with no electricity poised on the electrode.

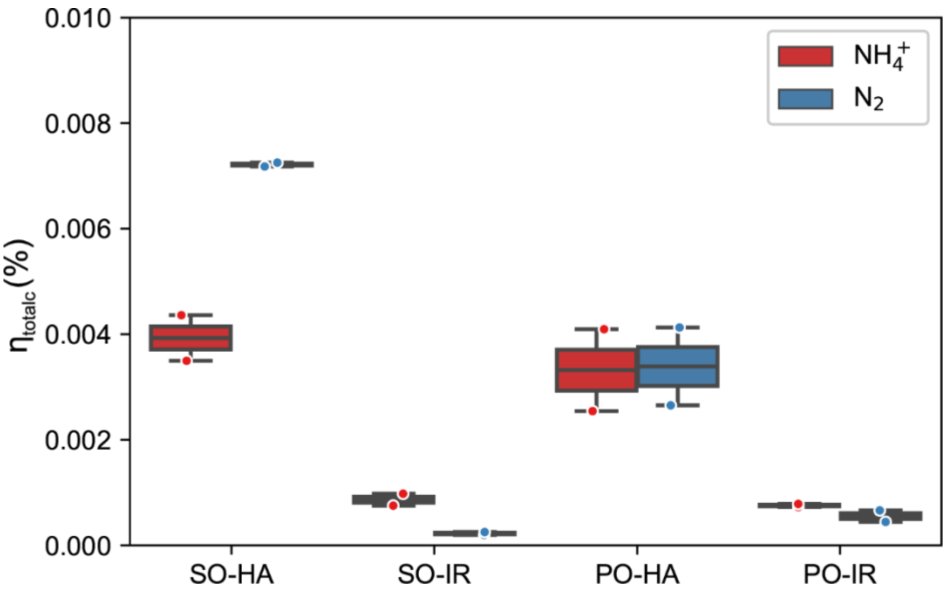

Supplementary Figure 7. Energy conversion efficiency from light. Data are from *n*=3 of independent experiments. SO: using electricity generated by a solar panel; HA: using halogen light as the light source. PO: using electricity from potentiostat as the electricity source; IR: using infrared light as the light source. NH_4^+: ammonium; N_2: dinitrogen gas.

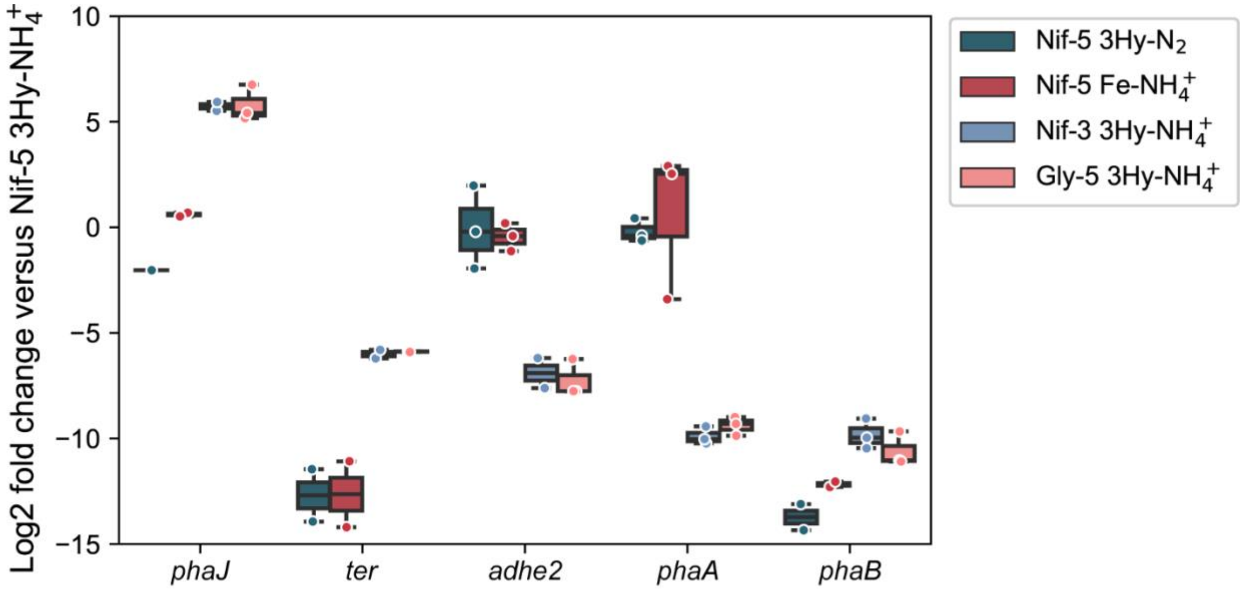

Supplementary Figure 8. mRNA log$_2$ fold change of butanol synthesis genes (*phaJ*, *ter*, *adhE*2, *phaA*, *and* *phaB*). Fold change was calculated using Nif mutant with 5-gene on 3Hy-NH_4^+ as the reference. We measured the total expression level of each enzyme. Accordingly, we did not distinguish between the different copies of the *phaA* and *phaB*. The fold changes were calculated using RT-qPCR with the comparative Ct method and used *clpX* and *recA* were used as internal standards. Data are from *n*=3 of independent experiments.

3Hy-N_2: using 3hydroxybutyrate as a major carbon/electron source and dinitrogen gas (N_2) as the nitrogen source. Fe-NH_4^+: using carbon dioxide (CO_2) as a carbon source, ferrous iron as an electron source, and ammonium (NH_4^+) as the nitrogen source. 3Hy-NH_4^+: using 3-hydroxybutyrate as major carbon/electron source and NH_4^+ as the nitrogen source. CO_2 was present in all conditions.

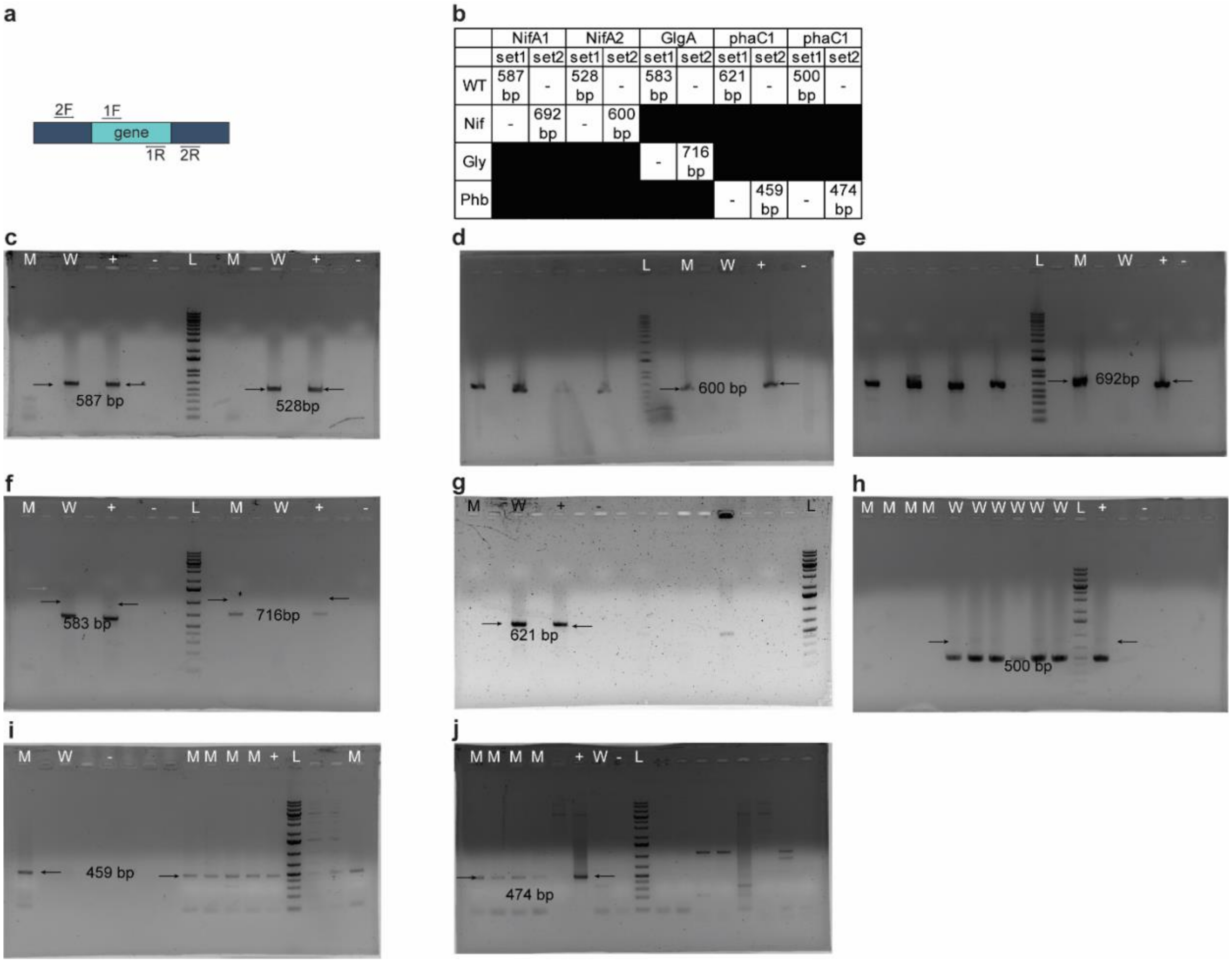

Supplementary Figure 9. PCR check for mutants. a. Schematic view of two different primer sets. **b**. Table of expected band sizes **c-e**: Nif mutant. **c**. To the left of the ladder: primer set 1 for *nifA*1 (Rpal_1624). To the right of the ladder: primer set 1 for *nifA*2 (Rpal_5113). **d**. To the right side of the ladder: primer set 2 for *nifA*1 (Rpal_1624). **e**. To the right side of the ladder primer set 2 for *nifA*2 (Rpal_5113). **f**. Gly mutant. To the left of the ladder: primer set 1 for *glgA* (Rpal_0386). To the right of the ladder: primer set 2 for *glgA* (Rpal_0386). **g-j**: Phb mutant. **g**. Primer set 1 for *phaC*1 (Rpal_2780). **h**. primer set 1 for *phaC*2 (Rpal_4722). **i**. Primer set 2 for *phaC*1 (Rpal_2780). **j**. Primer set 2 for *phaC*2 (Rpal_4722). Genomic DNA from WT or TIE- 1 mutant was used as a PCR template. Depending on the primer set, either WT(W) or mutant (M) genomic DNA was used as the positive control (+). Autoclaved Mili Q water was used as negative control (-). In panel **c-f**, L: Thermal Fisher 1kb plus DNA ruler. In panel **g-j**, L: GeneRuler 1kb plus DNA ruler. M: mutant; W: Wild type; +: positive control; -: negative control.

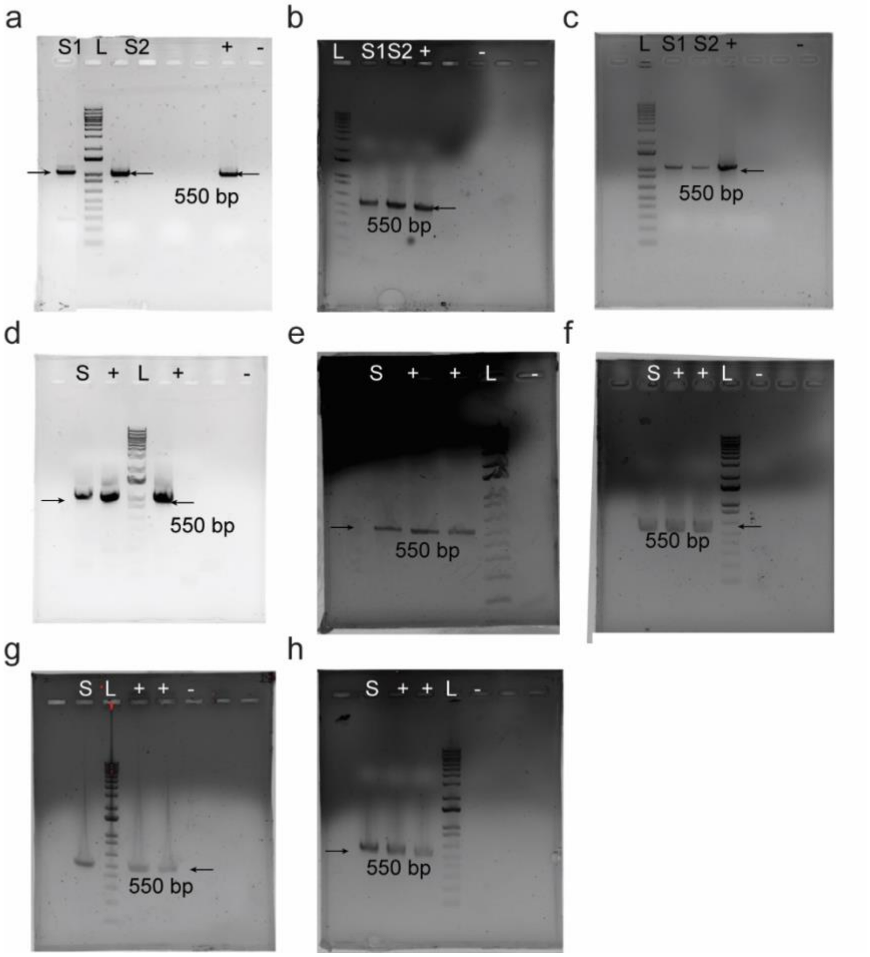

Supplementary Figure 10. PCR checks of inoculum for *n*-butanol cassettes. A-D: Inoculum for photoheterotrophic and photoautotrophic conditions using hydrogen or ferrous iron as electron donor **a.** WT. S1: WT with 3-gene cassette, S2: Wild Type with 5-gene cassette. **b.** Nif mutant. S1: Nif mutant with 3-gene cassette; S2Nif mutant with 5-gene cassette. **c.** Gly mutant. S1: Gly mutant with 3-gene cassette; S2: Gly mutant with 5-gene cassette. **d.** Phb mutant. S: Phb mutant with 3-gene cassette. **e-h:** Inoculums of Nif mutant with 5-gene cassette inoculum for photoelectroautotrophy. **e.** Photoelectroautotrophy using electricity from the solar panel under halogen light. Lane S: Nif mutant with 5-gene cassette. **f.** Photoelectroautotrophy using electricity from the solar panel under infrared light. S: Nif mutant with 5-gene cassette. **g.** Photoelectroautotrophy using electricity from potentiostat under halogen light. S: Nif mutant with 5-gene cassette. **h.** Photoelectroautotrophy using electricity from potentiostat under infrared light. S: Nif mutant with 5-gene cassette. A 550bp band is expected from both 3-gene and 5-gene cassettes. Miniprep from inoculum was used as a PCR template. A plasmid with either the 3-gene cassette or the 5-gene cassette was used as a

positive control. Autoclaved Milli Q water was used as negative control. L:1kb plus DNA ladder. S: sample; +: positive control; -: negative control.

Supplementary Table 1. Doubling time of *ΔnifA1ΔnifA2* using different carbon and nitrogen source.

Incubation Conditions	Doubling time (hr)	Doubling time (hr)
**3-Hydroxybutyrate + NH_4^+**	9.4 ± 2.5	11.2 ±1.8
**3-Hydroxybutyrate + N_2**	No growth	13.4 ± 1.2
**H_2 + NH_4^+**	218.5 ± 65.0	143.9 ± 63.7
**H_2 + N_2**	No growth	100.4 ± 3.2

Supplementary Table 2. Incubation combinations used in this study (except for photoelectroautotrophy).

Incubation condition		Nitrogen Sources	Electron Sources	Carbon Sources
1	Ac-NH_4^+	Ammonium (NH_4^+)	Acetate (Ac)	Acetate
2	Ac-N_2	Dinitrogen gas (N_2)		
3	3Hy-NH_4^+	Ammonium	3-hydroxybutyrate (3Hy)	3-hydroxybutyrate
4	3Hy-N_2	Dinitrogen gas		
5	H_2-NH_4^+	Ammonium	Hydrogen (H_2)	Carbon Dioxide (CO_2)
6	H_2-N_2	Dinitrogen gas		
7	Fe(II)-NH_4^+	Ammonium	Ferrous Iron (Fe(II))	
8	Fe(II)-N_2	Dinitrogen gas		

Supplementary Table 3. Incubation time of all cultures.

<table>
<tr><th></th><th>Ac-NH_4^+</th><th>Ac-N_2</th><th>3Hy-NH_4^+</th><th>3Hy-N_2</th><th>H_2-NH_4^+</th><th>H_2-N_2</th><th>Fe-NH_4^+</th><th>Fe-N_2</th><th>EU</th></tr>
<tr><td>WT+3</td><td colspan="4" rowspan="2">10 days</td><td colspan="4" rowspan="2">13 days</td><td rowspan="3">n.a.</td></tr>
<tr><td>WT+5</td></tr>
<tr><td>Nif+3</td><td rowspan="2">13days</td><td rowspan="2">16 days</td><td rowspan="2">13 days</td><td rowspan="2">16 days</td><td rowspan="2">16 days</td><td rowspan="2">19 days</td><td rowspan="2">16 days</td><td rowspan="2">19 days</td></tr>
<tr><td>Nif+5</td><td>10 days</td></tr>
<tr><td>Gly+3</td><td colspan="4" rowspan="3">10 days</td><td colspan="4" rowspan="3">13 days</td><td rowspan="3">n.a.</td></tr>
<tr><td>Gly+5</td></tr>
<tr><td>Phb+3</td></tr>
</table>

Ac: acetate; 3Hy: 3-hydroxybutyrate; H_2: hydrogen; Fe(II): ferrous iron; CO_2: carbon dioxide; NH_4^+ : ammonium; N_2: dinitrogen gas EU: photoelectroautotrophy WT-3: WT with 3-gene cassette; WT-5: WT with 5-gene cassette; Nif-3: *ΔnifA1ΔnifA2* with 3-gene cassette; Nif-5: *ΔnifA1ΔnifA2* with 5-gene cassette; Gly-3: *ΔglgA* with 3-gene cassette; Gly-5: *ΔglgA* with 5-gene cassette; Phb-3: *ΔphaC1ΔphaC2* with 3-gene cassette; n.d. :non-detectable; n. a. :not-applicable

Supplementary Table 4. Final optical density (OD_{660}) of all the construct under *n*-butanol producing conditions.

	Ac-NH_4^+		Ac-N_2		3Hy-NH_4^+		3Hy-N_2		H_2-NH_4^+		H_2-N_2		Fe-NH_4^+		Fe-N_2	
	ave	std	ave	std	ave	std	ave	std	ave	std	ave	std	ave	std	ave	std
WT+3	4.13	0.85	3.98	0.25	2.71	0.32	2.62	0.52	1.23	0.09	0.99	0.16	1.22	0.11	1.14	0.28
WT+5	5.14	0.49	1.52	0.32	3.61	0.19	1.44	0.08	1.67	0.16	1.53	0.02	1.89	0.05	1.73	0.10
Nif+3	4.33	0.21	2.20	0.04	2.46	0.21	0.86	0.03	1.72	0.15	1.78	0.10	1.17	0.31	1.15	0.07
Nif+5	3.30	0.44	1.12	0.02	2.92	0.17	1.05	0.03	1.85	0.05	1.20	0.19	1.73	0.06	0.74	0.05
Gly+3	7.46	1.72	3.96	0.87	7.24	0.97	4.04	0.72	1.04	0.32	0.78	0.11	0.83	0.09	0.77	0.05
Gly+5	4.86	0.60	2.47	0.31	3.26	0.18	1.86	0.08	1.41	0.07	1.54	0.27	1.56	0.11	1.44	0.08
Phb+3	3.93	0.45	1.99	0.68	5.78	0.37	1.68	0.45	2.02	0.09	2.00	0.45	1.40	0.06	2.05	0.15

ave: average, std: standard deviation Ac: acetate; 3Hy: 3-hydroxybutyrate; H_2: hydrogen; Fe(II): ferrous iron; CO_2: carbon dioxide; NH_4^+: ammonium; N_2: dinitrogen gas EU: photoelectroautotrophy WT-3: WT with 3-gene cassette; WT-5: WT with 5-gene cassette; Nif-3: *ΔnifA1ΔnifA2* with 3-gene cassette; Nif-5: *ΔnifA1ΔnifA2* with 5-gene cassette; Gly-3: *ΔglgA* with 3-gene cassette; Gly-5: *ΔglgA* with 5-gene cassette; Phb-3: *ΔphaC1ΔphaC2* with 3-gene cassette;

Supplementary Table 5. Doubling time of WT with pAB423 in media supplied with different amounts of *n*-butanol.

**Concentration of *n*-Butanol (mg/L)**	**0**	**2025**	**4050**	**8100**	**16200**
**Doubling time (hr)**	15.00	20.091	no growth	no growth	no growth

Supplementary Table 6. WT with pAB423 in media supplied with deferent amounts of acetone.

**Concentration of Acetone (mg/L)**	**0**	**1960**	**3920**	**7840**	**15680**
**Doubling time (hr)**	7.30	6.74	5.82	5.56	4.88

Supplementary Table 7. Incubation combinations used under photoelectroautotrophy.

Platforms		Nitrogen Sources	Light Sources	Electricity Sources
1	SO-HA-NH_4^+	Ammonium (NH_4^+)	Halogen light (HA)	Solar panel (SO)
	SO-HA-N_2	Dinitrogen gas (N_2)		
2	SO-IR-NH_4^+	Ammonium	Infrared light (IR)	
	SO-IR-N_2	Dinitrogen gas		
3	PO-HA-NH_4^+	Ammonium	Halogen light	Potentiostat (PO)
	PO-HA-N_2	Dinitrogen gas		
4	PO-IR-NH_4^+	Ammonium	Infrared light	
	PO-IR-N_2	Dinitrogen gas		

Supplementary Table 8. Strains used in this study.

Strains	Relevant genotypes of ***R. palustris*** **TIE-1**	Plasmid	Reference
AB437	Wild type (WT)	None	[1]
AB647	Δ*phaC1*Δ*phaC2* (Phb)	None	This study
AB133	Δ*nifA*1Δ*nifA*2 (Nif)	None	This study
AB145	Δ*glgA* (Gly)	None	This study
AB147	Wild type (WT)	pAB675	This study
AB153	Δ*phaC1*Δ*phaC2* (Phb)	pAB675	This study
AB149	Δ*nifA*1Δ*nifA*2 (Nif)	pAB675	This study
AB151	Δ*glgA* (Gly)	pAB675	This study
AB148	Wild type (WT)	pAB744	This study
AB150	Δ*nifA*1Δ*nifA*2 (Nif)	pAB744	This study
AB152	Δ*glgA* (Gly)	pAB744	This study

Supplementary Table 9. Primers for constructing and sequencing the plasmids used for generating mutants.

Primer name	Primer sequence
**Rpal_51131kbDnFwXbaI**	CATACTCTAGAAGCAGATCATCGTGGTGTCTTG
**Rpal_51131kbDnBamHIRev**	ATCAGGATCCCGCGGTCTCGGTGACCAGCTC
**Rpal_51131kbUpFwSacI**	TCATGAGCTCCAGAAGACGCTGGTGCTGAC
**Rpal_51131kbupRevXbaI**	GACTCTAGACATAGCTGGTCTCCATCGCTC
**Rpal_1624 1Kb Up SpeI Fw NifA**	TAGACTACTAGTCGTTTACAGCTCCGATCCGAATG
**Rpal_1624 1Kb Up NifA BamHI Rev**	ATACTAGGATCCGTCGGCTTCAGGACATGGTCG
**Rpal_16241kbDnXbaIFw**	CAGCTCTAGAATCATCAGACAAGGCGCGAC
**Rpal_16241kbDnBamHlIRev**	TCATGGATCCATTGGCAAGCGCATCACCCGGACC
**Rpal_2780 1Kb Up Fw**	CATATGACTAGTGAGTGTCCTTCAGCTTCTCCAGGA
**Rpal_2780 1Kb Up Rev**	CATATGGGATCCGAATCAACACTACAGTCCGGT
**Rpal_2780 1Kb Dn Fw**	CATATGGGATCCTGAACGACGCGCGGCGGCGAAGC
**Rpal_2780 1Kb Dn Rev**	CATATGCCCGGGACGGTGAGCACCGAATTCGCCTG
**Rpal_4722 1Kb Up Fw**	CATATGGCGGCCGCCGCGTGTCGTCTCAGCATTGCG
**Rpal_4722 1Kb Up Rev**	CATATGGGATCCCATCACCTCGTCGCGGCCGTC
**Rpal_4722 1Kb Dn Fw**	CATATGGGATCCTGAGCGGTCTGCGGCAACGCCGC
**Rpal_4722 1Kb Dn Rev**	CATATGCTGCAGTGGCCGACGACACCAACGAGCT
**Gly (Rpal_0386) UP XbaI F**	GCTATATCTAGAAGCGCAACGAGAGCTTCGACATTCTGCC
**Gly (Rpal0386) UP BamHI R**	ATATATGGATCCGCGTCGAGCTTCTCGATCATCGC
**Gly (Rpal_0386) DN BamHI F**	ATATATGGATCCCAAGCTGCGCACATCATCCCA

**Gly (Rpal_0386) DN XhoI R**	GCATATCTCGAGTCTGATCATGGAGCCGGCTTG
**Seq gly (Rpal_0386) F**	GAGAACACTGTGGTGCT

Supplementary Table 10. Primers used for checking the mutants.

Primer name	Primer sequence
PCR check Rpal_5113 set 1 F	GAGTGCTGACCTGAGCGAATAG
PCR check Rpal_5113 set 1R	CACTTGGTCGCGTCGATCACATAG
PCR check Rpal_5113 set 2 F	CGTTCGCACTTCCGGATGGAC
PCR check Rpal_5113 set 2 R	CTTGATGGTCTGGTTGCCGCCGAC
PCR check Rpal_1624 set 1 F	GAACCAGCTCGCGATCCATCTCAG
PCR check Rpal_1624 set 1R	GCCACGATGTAGACTTCCTGTGCCTTG
PCR check Rpal_1624 set 2 F	GAGCACCTTCTGGGCGAGCACGATC
PCR check Rpal_1624 set 2 R	ATGCCGATGGCCCAAAATTCCCG
PCR check Rpal_2780 set 1 F	CTCGCAACAATCGTCGCACTC
PCR check Rpal_2780 set 1R	AAGGATTGGCCTATACCG
PCR check Rpal_2780 set 2 F	CTTCCAGAACGAAATCATGCAGCTC
PCR check Rpal_2780 set 2 R	GGTGGCGTCGGAATTCCAGT
PCR check Rpal_4722 set 1 F	ATCCGCGGCTTGAGCAAGG
PCR check Rpal_4722 set 1R	TTGGCATCCCATTCCACG
PCR check Rpal_4722 set 2 F	GTCAAACTTCGCCCTCACCAATC
PCR check Rpal_4722 set 2 R	CGAGGCAATAGCCGACCG
PCR check Rpal_0386 set 1 F	TCACTTCGACAAGTCGTGCC
PCR check Rpal_0386 set 1 R	TAATCGTGACGATGGTCACC
PCR check Rpal_0386 set 2 F	AGGTCCACAGCTTCAACGAG
PCR check Rpal_0386 set 2 R	GTGTCGATGCCGTTGAGGAT

Supplementary Table 11. Plasmids used in this study.

Plasmid	Construct	Reference
pAB423	Empty Vector	[2]
pAB675	*phaJ*, *ter*, *adhE2* in pAB423	This study
pAB744	*phaJ*, *ter*, *adhE2*, *phaA*, *phaB* in pAB423	This study

Supplementary Table 12. Primers for constructing the 3-gene and the 5-gene cassette.

Primer name	Primer sequence
1F	ATCGAATTCCGCTAGCTTCACGCTGCCGCA
12F	GACCCGGCGTTTGCGGCGACCACGGCGTTC
23F	GTCGCGCATCACCGCCGCCTTCGGCTACGG
34F	ACTCGTATATCGGCCCGGAAGCGACCCAGG
45F	GACCGCATCTAAATGCATGCAGGATGAGGA
56F	GGACGCCGCCGTGAAGGCCGGCGCGCCGAA
67F	TGCAGTCCGTCGAGAAGTCGGAGCTGTTCA
78F	CTGTTCAAGCTGGGCTACGTCAACAAGATC
89F	CCACGCGATCGAGGCGTATGTGTCGGTGAT
12R	GAACGCCGTGGTCGCCGCAAACGCCGGGTC
23R	CCGTAGCCGAAGGCGGCGGTGATGCGCGAC
34R	CCTGGGTCGCTTCCGGGCCGATATACGAGT
45R	TCCTCATCCTGCATGCATTTAGATGCGGTC
56R	TTCGGCGCGCCGGCCTTCACGGCGGCGTCC
67R	TGAACAGCTCCGACTTCTCGACGGACTGCA
78R	GATCTTGTTGACGTAGCCCAGCTTGAACAG
89R	ATCACCGACACATACGCCTCGATCGCGTGG
10R	CGATCGATCGATCGATCTGCAGCTCCAAAA
910F	TCTACAACACCCTCGACAAGATGAGCGAGC
910R	CGATCGATCGATCGATCTGCAGCTCCAAAA
But XbaI NdeI F	CCGAGTCTAGACGTTTCATATGTCCGC
But EcoRI R	GCGCGAATTCTTAGATGCGGTCGAAGC
But EcoRI F	GCATGAATTCAGGATGAGGATCGTTTCGCATGAAGGT

But KpnI R	GTTAGGTACCGATCGATCGATCCATCTGCAGCTCC
phaA HindIII F	ATCGGCAAGCTTCTAACCAGGAGATGTCCATGTCGGA
phaB PstI R	ATATCTGCAGTCAAACCATGTATTGGCCGCCGTTGATGGTGA

Supplementary Table 13. Primers for sequencing the 3-gene and the 5-gene cassette.

Primer name	Primer sequence
But seq 2	GCGAGGACAAGCCAATCGCCACCCTCACCACCCGCATC
But seq 3	GCCTACTCGTATATCGGCCCGGAAGCGACCCAGGCCCTC
But seq new 2	AGTCACGGCCGAGGTGGAAG
But Seq new 3	GCGTCCTGAAGCCGTTCG
But Seq 4	CGGCATCATCGACCACGACGACAGCCTCGGCATCACCAAG
But Seq 5	CGGGCCATACCTCGTCGCTGTATATCGACAGCCAGAAC
phaA phaB Seq2	TGGTGCTGATGACCGCCAA
phaA phaB seq3	TGGGACGTGAGTTCGTTCGA

Supplementary Table 14. Specific oxidation state and number of electrons required per mole of *n*-butanol.

**Carbon source**	**The oxidation state of carbon in carbon source**	**The oxidation state of carbon in *n*-butanol**	**Mole carbon needed per mole *n*-butanol**	**Electron needed per mole *n*-butanol**
Acetate	0	-2	4	8
3-hydroxybutyrate	-0.5	-2	4	6
Carbon dioxide	4	-2	4	24

Supplementary Table 15. Primers for RT-qPCR.

Primer name	Primer sequence
qPCR phaJ F	CCTTTAAGCTGCCGGTGTTC
qPCR phaJ R	GTGGTGAGGGTGGCGATT
qPCR ter F	GCGCAACAATATCTGCCTGA
qPCR ter R	TGATGCGCTTCTTGGTGTAC
qPCR adhe2 F	ACCTGCTGTACGAGTATCCG
qPCR adhe2 R	CAGCTTCGGGAAATTGCAGA
qPCR phaA F	GGATGCCTTCAACGGTTACC
qPCR phaA R	CGCGAATTCATCCTGCTGAT
qPCR phaB F	TTTGAATTTCTCCGCTGCCG
qPCR phaB R	GGTATCGGTGCAGCAATCAG
TIE-1recAqRT-PCRFor	ATCGGCCAGATCAAGGAAC
TIE-1recAqRT-PCRRev	GAATTCGACCTGCTTGAACG
TIE-1clpXqRT-PCRFor	GGAGATCTGCAAGGTTCTCG
TIE-1clpXqRT-PCRRev	CCGCTTGTAGTGATTGTGGA
Km qPCR_F	CTCGTCCTGCAGTTCATTCA
Km qPCR_R	AGACAATCGGCTGCTCTGAT

Supplementary References:

1 Jiao, Y., Kappler, A., Croal, L. R. & Newman, D. K. Isolation and characterization of a genetically tractable photoautotrophic Fe(II)-oxidizing bacterium, *Rhodopseudomonas palustris* strain TIE-1. *Applied and Environmental Microbiol*ogy **71,** 4487-4496, (2005).

2 Katzke, N. et al. A novel T7 RNA polymerase dependent expression system for high-level protein production in the phototrophic bacterium *Rhodobacter capsulatus*. *Protein Expression and Purification*, **69,** 137-146, (2010).
