# Supplementary material for: n-Butanol production by Rhodopseudomonas palustris TIE-1
Source: Commun Biol. 2021 Nov 3;4:1257. doi: 10.1038/s42003-021-02781-z (PMC8566592; doi:10.1038/s42003-021-02781-z)
Supplement: Supplementary file 2 — Description of Additional Supplementary Files [file 42003_2021_2781_MOESM2_ESM.pdf]

Description of Additional Supplementary Files

File name: Supplementary Data 1.
Description: Source data.

File name: Supplementary Data 2.
Description: p-value tables.

File name: Supplementary Data 3.
Description: Effective size tables.
